# Experiences with COVID-19 economic relief measures among low-wage worker families: a qualitative study

**DOI:** 10.1186/s12889-024-20816-y

**Published:** 2024-11-29

**Authors:** Caitlin E. Caspi, Maria Gombi-Vaca, Curtis Antrum, Salma Gudaf, Molly De Marco, Emily Welle, Brett Sheppard, Kristi Fordyce, Rebekah Pratt

**Affiliations:** 1 Rudd Center for Food Policy and Health, University of Connecticut, 1 Constitution Plaza, Hartford, CT 061032 USA; 2 Department of Allied Health Sciences, University of Connecticut, 358 Mansfield Dr, Storrs, CT 06269 USA; 3 Center for Health Promotion & Disease Prevention, University of North Carolina at Chapel Hill, 1700 M.L.K. Jr Blvd #7426, Chapel Hill, NC 27514 USA; 4 Department of Nutrition, Gillings School of Global Public Health, UNC-CH, 135 Dauer Dr, Chapel Hill, NC 27599 USA; 5 Department of Family Medicine and Community Health, University of Minnesota, 717 Delaware St. SE, Minneapolis, MN 55445 USA

**Keywords:** Social determinants of health, COVID-19, Economic stability, Food insecurity, Housing stability

## Abstract

**Background:**

Economic stability is a core social determinant of health and a necessary condition for maintaining food security, housing stability, and both physical and mental health. Using a qualitative approach, we identified barriers, facilitators, and participant perceptions about utilizing these relief measures. This study aimed to understand experiences with COVID-19 economic relief measures among low-wage worker households with children during the COVID-19 pandemic.

**Methods:**

The study conducted semi-structured qualitative interviews from low-wage workers in households with children in two U.S. cities in 2022 (*n* = 40). The sample was recruited from a larger study which included survey measures of demographics and receipt or utilization of a range of relief measures with both broad and narrow eligibility criteria (e.g., Economic Impact Payments, expanded Supplemental Nutrition Assistance Program (SNAP), Pandemic Electronic Benefit Transfer (P-EBT), expanded Child Tax Credit, expanded unemployment insurance, and the eviction moratorium). A thematic analysis of 40 interviews was conducted using a constructivist approach to grounded theory, from which barriers, facilitators, and participant perceptions were identified.

**Results:**

Interviews identified burdensome administrative processes, administrative errors, and a lack of information as barriers to access among those who were eligible; automatic processes for distributing benefits was identified as a facilitator. Participants expressed positive perceptions about benefits, mixed with some ambivalence about the need to receive them and concern over their discontinuation.

**Conclusions:**

A segment of the population at risk of economic instability identified both barriers and facilitators to receiving an array of economic relief measures during the COVID-19 pandemic. Findings suggest that providing automatic processes for enrollment and reliable information streams for learning about benefits can bolster benefit receipt among those at risk of economic instability. These findings can contribute to the base of knowledge for policymakers involved in responding to the next public health emergency.

**Supplementary Information:**

The online version contains supplementary material available at 10.1186/s12889-024-20816-y.

## Introduction

The start of the COVID-19 pandemic was a time of social and economic upheaval. While disruptions occurred across the socioeconomic spectrum, households with children, racial and ethnic minorities, and those earning lower wages faced acute risks associated with the pandemic itself, as well as pandemic mitigation strategies. Job losses were concentrated in low-wage employment sectors [1]. Abrupt losses of earned income threatened people’s ability to pay rent and maintain stable housing [2]. For lower income households with children, school closures not only posed a childcare crisis, but also posed challenges maintaining food security for those relying on the United States Department of Agriculture’s (USDA)’s school meal programs [3]. Economic disruptions during the pandemic were more likely among racial/ethnic minorities [4]. Such patterns of economic disruption also had the potential to exacerbate existing inequalities in physical and mental health [5]. Indeed, Non-Hispanic Black and Hispanic communities and low socioeconomic populations were more vulnerable to COVID-19 outcomes, such as infection, hospitalizations and death [6, 7], as well as psychological distress [8].

To respond to the COVID-19 crisis and prevent a cascade of downstream consequences on health and wellbeing, the U.S. federal government rolled out multiple economic relief measures starting in March 2020. Some relief measures were aimed at relatively narrow segments of the population who were particularly economically vulnerable. For those experiencing job loss, the 2020 Coronavirus Aid, Relief, and Economic Security Act offered an unprecedented temporary increase in weekly unemployment benefits, as high as $600 in the first months of the pandemic [9]. Other relief measures included expansions and flexibilities of means-tested federal food assistance programs. As early as March 2020, the Families First Coronavirus Response Act allowed the USDA to issue Emergency Allotments, which increased Supplemental Nutrition Assistance Program (SNAP) benefits to the maximum amount for a household’s size [10, 11] for states that opted in. This action was followed by a temporary increase in the maximum SNAP benefit amount by 15% in 2021 [11]. Then, in April 2021, the USDA authorized additional Emergency Allotments of up to $95 per household, for the first time increasing benefits for households who previously were receiving the maximum benefit [12]. Meanwhile, the Pandemic Electronic Benefit Program (P-EBT) was launched at the start of the pandemic, offering additional benefits during school closures to parents of children who were eligible for school meals [13]. These benefits were meant to substitute for free and reduced-price meals offered when schools were open. State-specific temporary flexibilities in documenting income eligibility for these food assistance programs had the potential to increase participation [11].

Other economic relief measures were offered to a broader swath of the population. Three Economic Impact Payments (“stimulus checks”) were authorized from March 2020 to March 2021. They offered a total of $3,200 per adult and $2,500 per qualifying child [14] to nearly all low-and-middle income households who had filed taxes in the previous year. Similarly, the expanded Child Tax Credit [15] was a provision in the March 2021 American Rescue Plan Act. The credit not only increased the amount of the Child Tax Credit, but taxpayers were able to receive advance monthly payment advances of half of their credit amounts during the second half of 2021. Other policies provided indirect protection against acute economic disruptions. A key example is the early federal ban on housing evictions and the subsequent CDC-issued eviction moratorium effective through July 2021 [16, 17]. These housing policies, while not providing direct cash payments, were meant to provide broad protections against loss of housing. As eviction moratoria phased out, the federal government also offered temporary supports for those struggling to pay rent, including over $46 billion in funding through the Consolidated Appropriations Act and the American Rescue Plan to support Emergency Rental Assistance (ERA) programs administered at the state level [18]. These supports were layered upon other federal subsidies, state programs, and local supports that varied across localities [19, 20].

Preliminary evidence suggests that supports meant to protect people from economic disruptions associated with the pandemic were limited or inconsistent in their reach, even for those relief measures with broad eligibility [21–23]. Uneven patterns of assistance receipt may have contributed to persistent food insecurity and housing instability, as well as disparities in the economic, social, and health consequences of the pandemic era [4]. The aim of this study was to use a qualitative approach to understand barriers, facilitators, and participant perceptions about accessing these benefits in a sample of low-wage worker households with children in two U.S. cities.

## Methods

A sample of 40 participants who completed qualitative interviews was drawn from a parent study of low-wage worker households with children, the Wage$ study [24, 25].

### Parent study sample

The interview sample was drawn from a larger sample of adults participating in a parent study (Wage$). The Wage$ study was a longitudinal cohort study of low-wage workers in two cities (Minneapolis, Minnesota (MN), and Raleigh, North Carolina (NC) in which participants were followed from 2018 to 2022. The parent study had the primary aim of assessing the effect of an incremental minimum wage increase in Minneapolis on diet-related outcomes [24]. In 2018, participants were recruited for the Wage$ study from the community and gave informed consent to complete annual measures through 2022. They were eligible for the study if they: [1] were 18 years old or older [2], worked at least 10 h a week at a wage of less than or equal to $11.50/hour in Minneapolis/Raleigh OR were employed at that wage within the last six months and were currently seeking work in Minneapolis/Raleigh [3], planned to serve in the workforce for at least five years [4], agreed to be contacted for follow-up, and [5] spoke English or Spanish. No new participants were recruited after the baseline period in 2018. Unless they formally withdrew from the study or were deceased, the research team attempted to reach participants each year through 2022, even if they had missed completing measures in one or more years.

### Interview sample recruitment

In the summer of 2022, the study team aimed to conduct 40 semi-structured qualitative interviews to assess access to and experience with COVID-19 relief measures with a subset of participants of the larger sample. Potential interview participants were screened while at their annual study visit to determine if they were eligible, based on having reported at least one child of at least three years of age in their household in the current year’s survey (i.e., households had at least one child before the start of the COVID-19 pandemic). If invited participants could not be reached for scheduling, or if they declined after initially expressing interest, the interview was marked incomplete, and the next eligible participant who had an appointment was invited. Invitations occurred on a rolling basis until 40 had been completed. A total of 53 participants were invited to reach the target of 40 interviews. Eight people invited to participate initially expressed interest, but were unable to be reached for scheduling (three in Minneapolis, five in Raleigh). Five other people invited to participate declined (three in Minneapolis, two in Raleigh).

After the first 26 interviews, we examined relief measure utilization among those who completed interviews to ensure that the interview sample included adequate participation in the relief measure categories on which the study focused. Across relief measures, participation among those in the interview subsample was equal or greater than participation among those in the larger survey sample, suggesting adequate interview representation of participants who had received different relief measures. Fourteen additional interviews were completed to reach 40.

### Interview procedures

Informed consent was obtained from participants prior to the start of the interview. Interview questions were open-ended. The current study focused on questions about which supports they received during the pandemic, the process of accessing benefits, and any recommendations for policy improvements. Opening and concluding questions to the interviews also asked participants to describe the direct impact of the COVID-19 pandemic, as well as the impact of these relief measures on their household’s health; themes relating to direct pandemic impact and policy impact are reported elsewhere. Interviews were designed to last approximately 45 min and participants received $50 for their participation.

### Survey measures

Understanding the demographic characteristics and exposure to COVID-19 era policies among those who were interviewed provides an important context for the interpretation of the thematic findings. To describe the characteristics and COVID-19 relief measure exposure of the 40 participants, we report participant data from the Wage$ survey (Supplementary File): respondent demographics (age, sex, race/ethnicity, bank account status); respondent employment status; household characteristics (household size, number of children in the household, household composition, household income, household food security status, household housing stability status), and geographic location (MN, NC). The household income variable was coded to reflect three categories: [1] $20,000 or less [2], $20,001 to $40,000 [3], More than $40,000. The household food security status variable was created by combining the responses from the 6-item USDA food security measure [26], with responses coded as follows: food security (0 or 1 affirmative responses), or food insecurity (2 to 6 affirmative responses). Household housing stability status was based on three survey measures in relation to participant’s experience in the last 12 months [27, 28]: number of places lived (0, 1, 2, 3 or more than 3); not having a steady place to sleep or slept in a shelter (yes/no); and not being able to pay the mortgage or rent on time (yes/no). Participants were defined as experiencing housing instability if they had a positive response to either yes/no question or if they had lived in 0 or 3 or more places.

Survey questions related to COVID-19 economic relief measures asked in relevant years about whether study participants had received support from each program. Questions were added or removed in April/May of each year prior to starting data collection in July (2020, 2021 and 2022). The research team consulted the parent study’s Community Advisory Boards in Minneapolis and Raleigh about emerging relief measures that would be most relevant to study participants during these periods. The time period was referenced was “during the stay-at-home order” in 2020 and “in the last 12 months” in 2021 and 2022, with response options including yes, no, and don’t know. Relief measures included those for which the target population for the relief measure was broad or the eligibility for the benefit had few restrictions: Economic Impact Payments (“stimulus check”) (2020–2021), the expanded Child Tax Credit (2021–2022), and eviction prevention (“also known as eviction moratorium”) (2021–2022). It also included relief measures for which the target population was narrow and for which receiving the benefit depended on income or other qualifying circumstances: SNAP benefit increases (2021–2022), P-EBT (“a second food benefit on your EBT card”) (2021–2022), and unemployment insurance (2020–2022).

### Data analysis

COVID-19 relief measure utilization was described by calculating the percent of the sample that ever reported “yes” to receiving each of the COVID-19 relief measures. Survey data from 2020, 2021, and 2022 was used for the sample of 40 participants to determine whether they had reported receiving each support in any year in which they had responded to the item on the survey.

For the qualitative analysis, completed interviews were recorded and transferred to a transcription software for review and cleaning by the study team. The interview transcripts were systematically coded by study staff using NVivo (QSR international). The analysis used a constructivist approach to grounded theory [29]. This approach allowed data to be organized into emergent themes, while also allowing for consideration of our study aim to identify experiences relating to access to relief. Constructivist approaches to grounded theory are less concerned with data saturation, and as such, the analysis focused on exploring the experiences and perceptions of participations who had received a wide range of relief measures. Two team members double coded data and engaged in an iterative process of reviewing emergent codes and building a consensus-based codebook (coding framework). This was followed by the creation of a data summary document, which two additional team members organized thematically. The team worked collaboratively to ensure a rigorous process of systematic review and consensus building for the identification of the final themes. Disagreements were discussed and reviewed to ensure the team collectively agreed on the emergent codebook and themes. Data was re-reviewed and discussed with the broader team to guide this process as needed. Finally, themes were organized into barriers, facilitators, and participant recommendations.

### Positionality

We note that our research team reflects the experiences and identities of study participants in some ways, but not others. For example, we report that some team members experienced economic hardship and had received public assistance during their lives, but among the investigator team, no one received need-based relief during the height of the COVID pandemic. As another aspect of social identity, we report that the interviewers included two Black females; coders included one Black female, one Black male and two white females. We acknowledge that the team’s experience and social identities had the potential to influence how we developed our research aims, shaped our interview guide, administered the interviews, as well as the analysis and interpretation of our results. It is possible that our team missed important questions to ask because of lack of personal experience with these policies. However, the study was conducted with the input of Community Advisory Boards in both Minneapolis and Raleigh that had been in place since the start of the parent study. These boards were diverse in terms of race/ethnicity and socioeconomic status. The investigator teams met regularly with the boards throughout the study; members of the board contributed to the development of the interview guide and in the interpretation of the findings.

## Results

Characteristics of respondents in the interview samples are presented in Table 1. The sample was mostly female (85%), and there was a racial/ethnic mix of 70% Non-Hispanic Black, 22.5% Non-Hispanic White, 5% Hispanic, and 2.5% Non-Hispanic Other. In this sample, 60% reported food insecurity, 62.5% reported housing instability, and 55% reported no bank account. The average household size was 4.2 (SD = 1.6), and the average number of children in the households was 2.3 (SD = 1.1). The prevalence of households that received each COVID-19 economic relief support is shown in Table 2. The reported prevalence of benefit utilization among those responding to survey questions in any year ranged from 100% for Economic Impact Payments to 27.5% for the eviction prevention.

**Table 1:** Characteristics of respondents and households in the survey sample and in the interview sample (Minneapolis, MN; Raleigh, NC; Wage$, 2022)

	Interview sample	
	n (%)	
Sex	40	
Male	6	(15.0)
Female	34	(85.0)
Non-binary	0	(0.0)
Age	40	
< 30 years-old	8	(20.0)
30–39 years-old	21	(52.5)
40–49 years-old	6	(15.0)
50–59 years-old	4	(10.0)
60 years or older	1	(2.5)
Race/ethnicity	40	
Hispanic	2	(5.0)
Non-Hispanic White	9	(22.5)
Non-Hispanic Black	28	(70.0)
Non-Hispanic Other	1	(2.5)
Employment status	40	
Employed	33	(82.5)
Not employed	7	(17.5)
Bank account status	40	
Has a bank account	18	(45.0)
Does not have a bank account	22	(55.0)
Geographic location	40	
Minneapolis, MN	20	(50.0)
Raleigh, NC	20	(50.0)
Household composition	40	
Single adult w/ children	14	(35.0)
Multi-adult w/ children	26	(65.0)
Household income	40	
<$20 K Income	22	(55.0)
>$20–40 K Income	15	(37.5)
>$40 K Income	3	(7.5)
Housing stability status	40	
Housing stable	15	(37.5)
Housing unstable	25	(62.5)
Food security status	40	
Food secure	16	(40.0)
Food insecure	24	(60.0)

**Table 2:** Frequency of households with children that reported receiving COVID-19 economic relief measures in any year in which they completed survey measures (2020 to 2022) (Minneapolis, MN; Raleigh, NC, Wage$)

			Interview sample, *n* = 40	
**COVID-19 relief measures**	N Responded to item at any time point *N*		N (%) responding Yes to item at any time point *N (%)*	
Economic Impact Payments	37		37 (100.0%)	
P-EBT	40		34 (85.0%)	
SNAP benefit increases	40		33 (82.5%)	
Child Tax Credit	40		26 (65.0%)	
Unemployment insurance	39		22 (56.4%)	
Eviction moratorium	40		11 (27.5%)	

Qualitative fundings on the perceptions and experiences of participants with COVID-19 economic relief measures are presented below and are organized into three overarching themes. Sub-themes and illustrative quotes are integrated under each overarching theme. The three themes were utilization barriers (with the sub-themes of narrow eligibility criteria, burdensome or erroneous administrative processes, and a lack of public information); facilitators for receiving relief measures (with the sub-themes of automatic payments and information networks); and participant perceptions towards benefits (with the sub-themes of temporary changes, fear of discontinuation, gratitude, and skepticism).

### Utilization barriers

The major barriers cited by participants in accessing relief measures were related to those measures with narrow eligibility criteria, administrative processes that were burdensome or mishandled, and a lack of public information. For means-tested benefits like SNAP, income requirements and strict eligibility cutoffs were a commonly cited challenge. Several low-wage workers earned too much income to qualify for food assistance, but at the same time, it was clear that many were still struggling to make ends meet. One participant walked through the reality of her living circumstances, explaining why SNAP eligibility requirements posed a challenge for her household.

*“I make $41*,*000 a year. Although my household contains myself*,* a nine-year-old*,* a 22-year-old*,* and a 19-year-old. The 22-year-old and the 19-year-old are considered adults. So*,* whether they have a job or not*,* even though I’m the one taking care of them*,* they’re not included in my household when it comes to deciding if I qualify for something… even though really my household size is me and three kids*,* not one.” (Minneapolis P01)*.

Beyond income requirements, child custody arrangements added an additional complexity to benefit utilization in some cases. Children could only be claimed as dependents in one household in determining eligibility for SNAP, P-EBT, and the Child Tax Credit, which meant that in several households in which children were often present did not receive them. In the case of the Child Tax Credit benefit, one participant with shared custody of two children was not aware of the benefit.

*“No*,* I haven’t received any of that. And I don’t I don’t know. I’m not really sure about that- that information there. I don’t- if they did*,* I never seen a penny of it. At least…I didn’t in my home finance.” (Minneapolis P02)*.

Among the burdens on receiving benefits were income verifications and other administrative processes. Technical issues with enrollment and recertification were mentioned by several participants.

*“It’s like an obstacle just to get that because you got to peer qualify*,* you gotta be under the poverty level… and you can’t make this much money*,* and you got to send them proof of this*,* and proof of that*,* make sure the kid is yours*,* social security card. It’s like a lot.” (Minneapolis P03)*.

In the rapidly shifting early pandemic period, these administrative processes were not just burdensome to navigate, but were also sometimes faulty and could lead participants to miss out on benefits. In one case, a food assistance benefit card was sent to an old address. In another case, a participant’s income from a job that had ended was mistakenly counted as income, resulting in reduced SNAP benefits. Yet another participant found out that the unemployment office had given them too much in their unemployment checks. The participant was surprised to learn that they needed to pay back some of the unemployment benefits they had received. This ultimately placed an unexpected economic burden on them.

Delays or missed benefits also arose for some participants who could not receive automatic payments, for instance those who had not filed taxes. Participants without bank accounts faced a similar challenge when receiving Economic Impact Payments. One participant with a closed bank account received one Economic Impact Payment by check, but never received the other two.

*“I feel like everything just could have been sent as a check. And then like, if we did want it direct deposited, that we could have called, or did something online to make direct deposit… I feel like that [would have been] the smartest thing to do.” (Raleigh P01)*.

The challenges of these administrative problems were compounded by a lack of consistent information about benefits. Without a centralized place to get information about rapid policy changes in the midst of pandemic closures, participants recalled, at times, chasing information and struggling to contact anyone.

*“I called the hotline for the P-EBT*,* they said to call the school and I called the school. The school never answered the phone*,* so I ended up coming up to the school. The building was closed*,* so it’s like at this point*,* who I’m supposed to contact?” (Minneapolis P04)*.

Because of the lack of communication, benefits like P-EBT could come as a surprise, albeit a welcome one. Participants were clear about the high-stakes nature of information related to these benefits.

*“I think the information… You need to be specific. It needs to be spot on. We don’t have time for guessing games when it comes to everyone’s livelihood." (Raleigh P02)*.

Other relief measures came up during conversations with participants, beyond those that were specifically probed. These included childcare assistance, utility assistance, and rental assistance. At the time the interviews were conducted in 2022, both Minneapolis and North Carolina were still issuing emergency rental assistance for those who accrued rental debt during the pandemic. Participants faced many of the same challenges with rental assistance programs as with other programs, including confusion about eligibility, administrative challenges, and a lack of information. One participant sought assistance at a shelter and was told that they would have to be homeless for two weeks before they could get assistance. Another participant discussed how they could not receive emergency rental assistance because they did not have enough money saved.

*“I was told that I didn’t qualify for rental assistance*,* because I didn’t have income saved*,* like my rent is $1295. And they’ll pay up to $600 of the rent. But since I didn’t have the income to pay the remaining balance of the rent*,* I was- I didn’t qualify for it*,* which I was confused about.” (Raleigh P03)*.

Long waits for the distribution of the rental assistance led to uncertainty around the distribution of the benefit and disputes with landlords and the courts. Eviction was rare, but several participants faced the threat of eviction or went through court proceedings even though they had sought rental assistance. These challenges reinforce the administrative and communication challenges noted for other relief measures, and may have been further compounded by the novelty and hurried deployment of these programs.

### Facilitators to receiving relief measures

Information from qualitative interviews demonstrated that it was in many cases easy to get the benefits they were eligible for, but that it depended on the program. The main facilitators for easy access that emerged were automatic or direct deposit methods and being part of an existing network of services for learning about relief measures. Many participants discussed how automatic or direct deposit processes based on tax filings, including those for Economic Impact Payments and Child Tax Credits, facilitated the timely receipt of benefits. In the case of food assistance programs, participants noted the ease of receiving additional benefits if they were already enrolled in SNAP or the school lunch program. Because P-EBT program eligibility was aligned with the school lunch program eligibility, participants described the ease of access if they were receiving school lunches.

*“I automatically got it*,* the P-EBT. I just applied for the lunch program years ago so I automatically got the P-EBT so it wasn’t hard to access.”(Raleigh P04)*.

Most COVID-19 relief measures were put into effect through rapid policymaking efforts and modifications to existing programs early in the pandemic. The swiftness of these changes had the potential to grant people rapid access to benefits, and in some cases, was successful in doing so. At the same time, communication of these policy and programmatic changes was haphazard and uneven. A facilitator for participants that supported relief access was receiving information through social services or through programs participants were already signed up for. For those already enrolled in food assistance programs, the pathway to receiving additional food assistance benefits (e.g., SNAP Emergency Allotments and P-EBT) was mostly described as straightforward.

*“Even with the increase in the benefits*,* they did a good job about letting people know also. They were sending out letters to everybody…They would send out text messages also to everybody to notify them about the P-EBT benefits*,* and everybody was able to get to it through there. And then with the EBT allotment*,* they were doing the same thing and that’s how I found out about that also.”(Minneapolis P05)*.

These findings illustrate a potential gap between those already receiving benefits and those newly eligible. In instances where people did not file taxes or were not already receiving a class of benefits with strict eligibility criteria, it was more challenging to benefit from these measures, especially given the rapidly changing circumstances of the pandemic.

### Participant perceptions towards benefits

The temporary nature of the support presented a challenge for participants. As one-time payments, benefits like the Economic Impact Payments did not have a long-term impact on participants’ financial standing.

*“The only thing that was easy was the stimulus check they gave you and the Child Tax Credit. But soon as they gave that to you*,* it was gone.” (Raleigh P03)*.

Even for support with more durability, like SNAP, the discontinuation of Emergency Allotments loomed. Participants often felt that they did not have enough time to prepare for the discontinuation of benefits.

*“No*,* it was like*,* it was basically like they were holding our hand along the way*,* and then all of a sudden pushed us down the hill and said*,* “Whoever makes it*,* makes it. Whoever doesn’t*,* tough…” (Raleigh P05)*.

Other participant comments centered around perceived deficits in benefit eligibility and benefit amount. In some instances, their hardship felt so intractable that the benefit amounts were perceived as inadequate.

*“It was like a catch up. It was like a pour into the cup to make it more to what’s already being sucked out of you if that makes sense. You got straws in different avenues for bills*,* so it didn’t help. It didn’t.” (Raleigh P06)*.

It was not uncommon for participants to have mixed feelings about the benefits they received. Even while many expressed gratitude for assistance during a time of upheaval, at times these relief measures were greeted with degree of skepticism, with one Minneapolis participant wondering if it was “*too good to be true*…” These sentiments were sometimes intertwined with long-standing negative perceptions of government assistance.

*“I don’t like anything being given to me. I hate when I have to reach out to organizations… because anything financial…when it comes to the government… comes back and somehow they get it back. Whether it be at tax time they get taxed on it or something. And so no*,* no*,* there was nothing that I felt I wish I got.” (Minneapolis P06)*.

In these instances, participants’ experience with COVID-19 relief measures may have been colored by previous encounters with benefits that were not always positive.

## Discussion

During the COVID-19 pandemic, most of our sample of low-wage worker households received multiple relief measures meant for both broad and targeted segments of the population. Participants noted a range of barriers, including administrative processes, administrative errors, and a lack of information. Meanwhile, automatic processes for benefit distribution and already being enrolled to receive some services facilitated their receipt.

For means-tested programs like SNAP and other food assistance programs, meeting income eligibility requirements is just one of many known hurdles to participation [30]. Interviews highlighted how such low income thresholds for food assistance prevented some from accessing benefits, even when they were in need. This occurred in households where participants were working but made slightly too much to qualify for food assistance, or in households where young adults who could not be considered dependents were not contributing enough income for the household to make ends meet. Other barriers to benefit receipt that were evident even before the pandemic also persisted during this period. Threats posed by fluctuations in benefits and administrative burdens have been well-documented in the previous literature around SNAP participation [31–33]. It was not uncommon for Wage$ study participants to hold negative perceptions of government assistance before the pandemic [34], and ambivalence around receiving assistance persisted for some during the pandemic.

Aspects of the pandemic environment were different and exacerbated challenges in issuing benefits. Administrative agencies had to make adaptations to the program expansions and flexibilities offered during COVID-19; challenges with these adaptations may have accounted for some of the errors that participants reported [35]. While the rapid deployment of relief measures through existing programs provided the structure for expanded benefits like SNAP and made it seamless for some, at the same time, it may have compounded difficulties in access or feelings of mistrust for others who did not qualify. The expansion of existing measures meant that there were many systems to navigate, and may have complicated engagement with them for some households. Moreover, despite expanded benefits, during the pandemic period many SNAP participants continued to face unmet food needs [36, 37].

Beyond challenges with food security, it is notable that more than half of participants interviewed were experiencing housing instability. While eviction is rare and eviction moratoria offered some protection during the early pandemic period, it was common for participants to express worry about paying the rent or the consequences of falling behind on rent. Those facing housing instability may have been especially attuned to the receipt of flexible cash benefits such as the Child Tax Credit and Economic Impact Payments, which could be used for rental or housing payments. Challenges with stable housing can interfere with benefit receipt due to the administrative burden of maintaining benefits. Accounts of participants who had benefit checks mailed to the wrong address are a straightforward example, but households who move frequently face other challenges in maintaining benefits as well. For example, families who frequently relocate may miss notifications about when recertification for SNAP benefits is required. Recent research suggests that eviction is associated with a marked decline in SNAP [38]. These patterns point to the need for additional supportive services and administrative flexibilities to ensure the continuation of benefits among those who are housing insecure.

While the current study of low-wage worker families was conducted in two different U.S. cities, site differences between Minneapolis and Raleigh did not emerge as a salient theme in our analysis. This is perhaps surprising, since the receipt of federal relief measures took place during a global pandemic in which conditions differed by locality, including the timing and severity of COVID-19 waves, government-issued mitigation strategies, and other local supports that were offered. Experiences of economic relief measures may have been shaped by these variables. Moreover, relief measures, while federally authorized, were not implemented uniformly across all 50 states [11, 35]. However, the major COVID-19 era relief measure implementation and COVID-19 mitigation strategies were quite similar across the two study sites in key ways, including the timing and duration of stay at home orders [19]. Both Minnesota and North Carolina opted to continue issuing Emergency Allotments until the last month possible in 2023, whereas other states ended Emergency Allotments sooner [10]. Both states offered Emergency Rental Assistance programs following the end of the federal eviction moratorium [18]. The similarity across sites may be due to the relatively small sample size and the focus on federally supported relief measures.

### Limitations

This qualitative study has a number of limitations which should be considered. First, the findings may not be representative of all identities or experiences during the COVID-19 pandemic. For example, our participants came from only two states in the US which implemented federal COVID-19 relief measures in a largely similar way. Some other states ended Emergency Allotments early, and participants in those states may have reflected on them differently given the different duration. The sample also consisted of a greater number of female participants than male participants, and a greater proportion of interview participants were female than in the Wage$ cohort [24]. While drawing a sample from a pre-existing study provided a unique opportunity to recruit from a group of individuals who had known, complex financial needs prior to the pandemic, it is not necessarily representative of experiences of those with lesser financial need.

As another limitation, the assessment of benefit receipt was challenging for both the interview and survey data components. Relief measures and policy changes were often complex and difficult to understand for respondents. It was not a requirement to participate in interviews that the respondent be the head of household or the primary manager of finances in the household. This meant that participants did not always know policy or program names, which may have led to misreporting from survey data, or incomplete narratives during qualitative interviews. The interpretation of findings should be considered in this context.

## Conclusion

The swift deployment of multiple relief measures during the COVID-19 pandemic meant that most low-wage worker households received a patchwork of economic relief during this period. These benefits had the potential to mitigate serious health risks associated with economic disruption, including eviction and food insecurity. Participants’ experiences accessing these benefits were mixed. While broad eligibility and automatic distribution of some COVID-19 relief measures generally promoted participation, narrative accounts from an economically vulnerable segment of the population highlight numerous challenges in deploying economic relief during times of crisis, including strict eligibility for some benefits, administrative burdens that hindered access, and a lack of clear communication around complex policy changes.

## Electronic supplementary material

Below is the link to the electronic supplementary material.

Supplementary Material 1

## Data Availability

The quantitative dataset analysed in the current study is secondary data not publicly available as it is being analysed for primary outcomes from that study, but will be made available from the corresponding author on reasonable request. The qualitative dataset is not publicly available due to the possibility of that participants could be identified by details contained in the full transcription.

## Abbreviations

SNAP: Supplemental Nutrition Assistance Program
P-EBT: Pandemic Electronic Benefit Transfer
USDA: United States Department of Agriculture
MN: Minnesota
NC: North Carolina
