# Supplementary material for: Experiences with COVID-19 economic relief measures among low-wage worker families: a qualitative study
Source: BMC Public Health. 2024 Nov 29;24:3330. doi: 10.1186/s12889-024-20816-y (PMC11606178; doi:10.1186/s12889-024-20816-y)
Supplement: Supplementary file 1 — Supplementary Material 1 [file 12889_2024_20816_MOESM1_ESM.pdf]

COVID-19 relief measures and food insecurity among low-wage worker families

Interview Guide

Thank you for joining us for an interview today. We are really pleased you are willing to share your experiences with us. In sharing with us, you are contributing knowledge that can only be gained through lived experiences. This knowledge is so important in understanding how the pandemic affected people, and what we should be doing to make conditions better for people. Do you have any other questions before we get started?

Today, we are interested in hearing about the different relief benefits or payments that people or families received during Covid-19.

1. To start with, I'd like to ask you to reflect on your experience during the Covid-19 pandemic. Can you share a little bit about your experience over the past three years, and how the pandemic impacted your work, household budget, income or expenses?

2. We appreciate your work in completing our previous study surveys. From those surveys we can see that in the past you accessed several policy supports (*Share those supports noted on previous surveys).* Is there anything else we should add to that list? Did you or your family receive any additional government support during Covid-19? *Prompt, as necessary: child tax credit, SNAP, pandemic EBT, stimulus checks, unemployment, housing eviction moratorium, health care subsidies?*

3. We'd now like to talk with you about those supports and how they have impacted you. When you think about the various supports you received, can you describe how easy you felt it was to access those benefits? *Prompt: Which ones were straightforward to access? Why do you think that was? Which ones were most challenging to access? Why do you think that was?*

4. What would have made it easier to access these supports for you? *Probe: Can you say more about why that would have been helpful?*

5. How did you learn about the support you accessed? *Prompt: From whom? From what sources?*

6. Other common supports or benefits that we haven't yet discussed today include (*mention supports not mentioned).* Have you heard about any of these other benefits? *Probe: Which ones, what do you know about those?*

7. Were there any benefits that you didn't receive, or applied for and didn't get? Can you share why you didn't get that/those benefits? *Probe: which ones, can you share more about why?*

8. Thinking back to the benefits you received, which onces led to changes in your household economic security? *Prompt: Which benefits helped your household budget*

the most, like your expenses and your income? In what ways were they helpful? Can you give an example?

9. How long-lasting or short-lived were these changes? *Prompt: Do things continue to be changed for your household?*

10. Which of the benefits you received led to changes in the ability to afford or access food for you? *Probe In what ways were they helpful, can you provide an example of that?*

11. How do you feel those benefits impacted the diet quality for you or your family? What about for the children in your household? Probe: *In what ways were they helpful, can you provide an example of that?*

12. In what other ways did the supports you received affect your health? *Probe: Can you share an example?*

13. When you think about the different supports you received that were related to Covid relief, which of those have now ended? *Probe: How have programs ending affected you or your family?*

14. What else would be helpful for us to know when thinking about how additional supports that were available during the pandemic impacted you or your family?

15. If you could give feedback to the people that created these policies or programs, what would you tell them?

Thank you for your time.

2018 (T1) Participant Information

Record ID __________________________________

Subject ID __________________________________

Please tell us about your background...

Date of study enrollment __________________________________

First Name __________________________________

Last Name __________________________________

Are you:
- ○ Male
- ○ Female
- ○ Non-binary

How old are you? __________________________________
(Years)

Are you Hispanic or Latino/a?
- ○ Yes
- ○ No

What is your race?
(Check all that apply)
- ☐ American Indian/Alaska Native
- ☐ Asian
- ☐ Black or African American
- ☐ Native Hawaiian or Other Pacific Islander
- ☐ White
- ☐ Other

If you selected other, please specify: __________________________________

Were you:
- ○ Born in the U.S. or U.S. territories
- ○ Foreign born (not of U.S. parent or parents)
- ○ Born abroad to U.S. citizen parent or parents

If you were born outside the U.S., in what country were you born? __________________________________

What is you marital status?
- ○ Married/partnered
- ○ Single

Participant Details from Call Log

Name: [name]
Phone number: [phone]
Email: [email]

2018 (T1) Participant Survey_English

Please complete the survey below.

Thank you!

How many adults (age 18 or older) currently live in your household (including yourself)?	◯ 1<br>◯ 2<br>◯ 3<br>◯ 4<br>◯ 5 or more
How many children (under the age of 18) currently live in your household, including children who live there part-time?	◯ 0<br>◯ 1<br>◯ 2<br>◯ 3<br>◯ 4<br>◯ 5 or more

Please list the following information for each child living in the household:

Child 1
Age

________________ (Years)

Weight

________________ (Pounds (lbs))

Height

________________ (Inches)

Child 2
Age

________________ (Years)

Weight

________________ (Pounds (lbs))

Height

________________ (Inches)

Child 3
Age

________________ (Years)

Weight

________________ (Pounds (lbs))

Height

________________ (Inches)

Child 4
Age

________________ (Years)

Weight

________________ (Pounds (lbs))

Height

________________ (Inches)

Child 5
Age

________________ (Years)

Weight

________________ (Pounds (lbs))

Height	__________________ (Inches)
How did you determine the child or children's height or weight?	◯ Pediatrician or health care provider records<br>◯ School records<br>◯ Measured at home<br>◯ My best guess<br>◯ Other
If you selected other, please specify:	__________________
Are you registered to vote?	◯ Yes<br>◯ No
Did you vote in the last local election, like for mayor or city council member?	◯ Yes<br>◯ No
In the last year (12 months), was there a time when you were not able to pay the mortgage or rent on time?	◯ Yes<br>◯ No
In the last year (12 months), how many places have you lived?	◯ 0<br>◯ 1<br>◯ 2<br>◯ 3<br>◯ More than 3
In the last year (12 months), was there a time when you did not have a steady place to sleep or slept in a shelter (including now)?	◯ Yes<br>◯ No
What is the highest grade or level of school you have completed?	◯ Less than High School<br>◯ Some High School<br>◯ High School Diploma<br>◯ Associate/Technical Degree<br>◯ Some College<br>◯ Bachelor's Degree<br>◯ Graduate Degree
When did you complete this schooling?	__________________ (Year (YYYY))
Have you completed any of the following types of trainings/career development activities during the last year (12 months)? (Check all that apply)	☐ A training, workshop, seminar or professional event required by your employer<br>☐ A training, workshop, seminar or professional event not required by your employer<br>☐ Licensure or certification for your occupation<br>☐ A training, workshop, seminar or professional development event to help you change to a new occupation<br>☐ Other<br>☐ None of the above

If you selected other, please specify: ____

What was your annual household income (from all sources) last year, before taxes?

- ◯ Less than $5,000
- ◯ $5,001 to $10,000
- ◯ $10,001 to $20,000
- ◯ $20,001 to $30,000
- ◯ $30,001 to $40,000
- ◯ $40,001 to $50,000
- ◯ More than $50,000

In the last month (30 days) did you or anyone in your household receive any of the following? (Check Yes or No for EACH program)

	Yes	No	Not Sure
WIC (Women, Infants, and Children program)	◯	◯	◯
Food stamps (SNAP)	◯	◯	◯
Free or reduced price school lunch	◯	◯	◯
Minnesota Family Investment Program (MFIP)	◯	◯	◯
State housing subsidy (e.g., Bridges Housing Subsidy)	◯	◯	◯

How much did your household receive in SNAP benefits in the last month (30 days)?

◯ I do not receive any food stamps or SNAP benefits
◯ $1 - $25
◯ $26 - $50
◯ $51 - $75
◯ $76 - $100
◯ $101 - $150
◯ $151 - $250
◯ $251 - $500
◯ $501 - $750
◯ More than $750

Part B. Work and Finances

What is your average monthly income from wages (i.e. pay for the jobs you yourself work)?

- ◯ Less than $500
- ◯ $501 to $1,000
- ◯ $1,001 to $1,500
- ◯ $1,501 to $2,000
- ◯ $2,001 to $2,500
- ◯ More than $2,500

Do you have a physical health condition or disability that affects the number of hours of work you perform in a week?

- ◯ Yes
- ◯ No

Do you have mental health condition or disability that affects the number of hours of work you perform in a week?

- ◯ Yes
- ◯ No

Which one of the following best describes how well you are managing with money these days:

- ◯ Living comfortably
- ◯ Doing okay
- ◯ Just getting by
- ◯ Finding it difficult to get by

Compared to one year (12 months) ago, would you say that you (and your family living with you) are better off, the same, or worse off with money?

- ◯ Much better off
- ◯ Somewhat better off
- ◯ About the same
- ◯ Somewhat worse off
- ◯ Much worse off

Which of the following hardships did you or your family experience in the last year (12 months)? (Check all that apply)

- ☐ Had a medical procedure or health emergency
- ☐ I lost a job
- ☐ I had my hours reduced
- ☐ My partner/spouse lost their job
- ☐ My partner/spouse had their hours reduced
- ☐ Divorce/Separation
- ☐ Received a foreclosure or eviction
- ☐ Death of another income earner
- ☐ Lost transportation (vehicle repossessed or totaled, bus service cut etc...)
- ☐ Other
- ☐ None of the above

If you selected other, please specify:

____

Have you used any of the following services in the last year (12 months)? (Check all that apply)

- ☐ Pawn Shop
- ☐ Pay Day Loan
- ☐ Auto Title Loan
- ☐ Paycheck Advance Service
- ☐ Tax Refund Anticipation Loan
- ☐ Borrowed money from a friend, family or community member
- ☐ None of the above

If you were faced with a $400 emergency expense today, how would you most likely cover it?<br><br>(Check all that apply)	☐ Use money from recent paycheck or savings<br>☐ Borrow money from a friend, family or community member<br>☐ Put it on my credit card and pay it off over time<br>☐ Sell something you own<br>☐ Delay payment on other bills or expenses<br>☐ Pawn Shop<br>☐ Pay Day Loan or Paycheck Advance Service<br>☐ Auto Title Loan<br>☐ Tax Refund Anticipation Loan<br>☐ I do not know how I would cover this expense<br>☐ Other
If you were faced with a $500 emergency expense today, how would you most likely cover it?<br><br>(Check all that apply)	☐ Use money from recent paycheck or savings<br>☐ Borrow money from a friend, family or community member<br>☐ Put it on my credit card and pay it off over time<br>☐ Sell something you own<br>☐ Delay payment on other bills or expenses<br>☐ Pawn Shop<br>☐ Pay Day Loan or Paycheck Advance Service<br>☐ Auto Title Loan<br>☐ Tax Refund Anticipation Loan<br>☐ I do not know how I would cover this expense<br>☐ Other
If you were faced with a $600 emergency expense today, how would you most likely cover it?<br><br>(Check all that apply)	☐ Use money from recent paycheck or savings<br>☐ Borrow money from a friend, family or community member<br>☐ Put it on my credit card and pay it off over time<br>☐ Sell something you own<br>☐ Delay payment on other bills or expenses<br>☐ Pawn Shop<br>☐ Pay Day Loan or Paycheck Advance Service<br>☐ Auto Title Loan<br>☐ Tax Refund Anticipation Loan<br>☐ I do not know how I would cover this expense<br>☐ Other
In the past year, have you put off paying a bill because you didn't have enough money to pay it when it was due?	○ Yes<br>○ No
Do you have an account with a bank or credit union?	○ Yes<br>○ No

Part C. Accessing Food

What is the name and location of the store where MOST of the food for your household is purchased?

Name
Location (cross streets, nearby landmark)
City

____

Who usually shops for most of the food for your household?

(Choose more than one if the task is evenly split)

- ☐ Me
- ☐ Spouse/Partner
- ☐ Child/Children
- ☐ Other adult in the home
- ☐ Other

If you selected other, please specify:

____

How do you usually get to the store where you get most of your food?

- ◯ Drive myself in a vehicle
- ◯ Family member/friends drive me in their vehicle
- ◯ Take public transportation (e.g. bus)
- ◯ Walk
- ◯ Uber, or other car service
- ◯ Other

If you selected other, please specify:

____

Please indicate how often each statement below was true for your household in the last year (12 months):

	Often true	Sometimes true	Never true
The food that we bought just didn't last and we didn't have money to get more.	◯	◯	◯
We couldn't afford to eat balanced meals.	◯	◯	◯

In the last year (12 months), did you or other adults in the household ever cut the size of your meal or skip meals because there was not enough money for food?

◯ Yes
◯ No
◯ Don't Know

How often did this happen?

◯ Almost every month
◯ Some months but not every month
◯ 1 or 2 months

In the last year (12 months), did you ever eat less than you felt you should because there wasn't enough money for food?

◯ Yes
◯ No
◯ Don't Know

In the last year (12 months), were you ever hungry but didn't eat because there wasn't enough money for food?

◯ Yes
◯ No
◯ Don't Know

Part D. Your Health

In general, would you say your health is:	◯ Excellent<br>◯ Very good<br>◯ Good<br>◯ Fair<br>◯ Poor
Are you currently pregnant?	◯ Yes<br>◯ No
What type(s) of health insurance/health coverage do you currently have?<br><br>(Check all that apply)	☐ Medicaid<br>☐ Medicare<br>☐ Health insurance plan offered through your employer<br>☐ Health insurance plan obtained through Healthcare.gov or MNsure (ACA Exchange)<br>☐ Veterans' Administration (VA) Health Benefits<br>☐ Indian Health Services (IHS) tribal and/or urban Indian health programs<br>☐ Uninsured<br>☐ Other
If you selected other, please specify:	__________________________________
When was the last time you visited a doctor, physician assistant or nurse in a clinic or medical office?<br><br>Do not include times you were hospitalized overnight or visits to the hospital emergency room.	◯ Within the last 12 months<br>◯ Within the last 1-2 years<br>◯ Within the last 2-5 years<br>◯ More than 5 years ago<br>◯ Never
In the last year (12 months), did you have an illness, injury, or condition that needed care right away in a clinic, emergency room, or doctor's office?	◯ Yes<br>◯ No
In the last year (12 months), when you needed care right away, how often did you get care as soon as you thought you needed it?	◯ Never<br>◯ Sometimes<br>◯ Usually<br>◯ Always
In the last year (12 months), not counting the times you needed care right away, did you make appointments for your health care at a doctor's office or clinic?	◯ Yes<br>◯ No
In the last year (12 months), how often did doctors or other health providers listen carefully to you?	◯ Never<br>◯ Sometimes<br>◯ Usually<br>◯ Always
In the last year (12 months), how often did doctors or other health providers show respect for what you had to say?	◯ Never<br>◯ Sometimes<br>◯ Usually<br>◯ Always

In the last year (12 months), how often did doctors or other health providers spend enough time with you?

◯ Never
◯ Sometimes
◯ Usually
◯ Always

Please check one of the boxes to indicate how strongly you agree or disagree for each statement.

	Strongly Disagree	Somewhat Disagree	Neutral	Somewhat Agree	Strongly Agree
I'm healthy enough that I really don't need health insurance.	○	○	○	○	○
Health insurance is not worth the money it costs.	○	○	○	○	○
I'm more likely to take risks than the average person.	○	○	○	○	○
I can overcome illness without help from a medically trained person.	○	○	○	○	○

Considering a 7-Day period (a week), how many times on the average do you do the following kinds of exercise for more than 15 minutes during your free time:

STRENUOUS EXERCISE<br>(Heart beats rapidly)<br>Examples: running, jogging, hockey, football, soccer, basketball, cross-country skiing, roller blading, vigorous swimming, vigorous bicycling	__________________<br>(_____ times/week)
MODERATE EXERCISE<br>(Not exhausting)<br>Examples: fast walking, baseball, tennis, easy bicycling, badminton, easy swimming, downhill skiing, recreational dancing	__________________<br>(_____ times/week)
MILD EXERCISE<br>(Minimal effort)<br>Examples: easy walking, yoga, fishing, bowling, golf	__________________<br>(_____ times/week)
How much time do you usually spend sitting or reclining on a typical day?<br><br>Include time spent sitting at work, at home, getting to and from places (i.e. in a car, bus or train).<br>Do not include time spent sleeping.	__________________<br>(Hours)
How much time did you spend last week playing video games?	__________________<br>(Hours)
What time do you usually go to bed (turn off the lights to sleep)?	◯ 1 AM ◯ 2 AM ◯ 3 AM<br>◯ 4 AM ◯ 5 AM ◯ 6 AM<br>◯ 7 AM ◯ 8 AM ◯ 9 AM<br>◯ 10 AM ◯ 11 AM ◯ 12 PM (noon)<br>◯ 1 PM ◯ 2 PM ◯ 3 PM<br>◯ 4 PM ◯ 5 PM ◯ 6 PM<br>◯ 7 PM ◯ 8 PM ◯ 9 PM<br>◯ 10 PM ◯ 11 PM ◯ 12 AM (midnight)

What time do you usually get out of bed?	◯ 1 AM ◯ 2 AM ◯ 3 AM<br>◯ 4 AM ◯ 5 AM ◯ 6 AM<br>◯ 7 AM ◯ 8 AM ◯ 9 AM<br>◯ 10 AM ◯ 11 AM ◯ 12 PM (noon)<br>◯ 1 PM ◯ 2 PM ◯ 3 PM<br>◯ 4 PM ◯ 5 PM ◯ 6 PM<br>◯ 7 PM ◯ 8 PM ◯ 9 PM<br>◯ 10 PM ◯ 11 PM ◯ 12 AM (midnight)
Please select one of the following:	◯ Current smoker ◯ Quit less than 12 months ago ◯ Quit more than 12 months ago ◯ Never smoked

Please indicate how often each statement below was true for you in the last month (30 days)

	Never	Almost Never	Sometimes	Fairly Often	Very Often
a.In the last month (30 days), how often have you felt unable to control the important things in your life?	○	○	○	○	○
b.In the last month (30 days), how often have you felt confident about your ability to handle your personal problems?	○	○	○	○	○
c.In the last month (30 days), how often have you felt that things were going your way?	○	○	○	○	○
d.In the last month (30 days), how often have you felt difficulties were piling up so high that you could not overcome them?	○	○	○	○	○

Part E. Dietary Screener Questionnaire

These questions are about foods you ate or drank during the past month, that is, the past 30 days. When answering, please include meals and snacks at home, at work or school, in restaurants and anyplace else.

During the past month (30 days), how often did you eat hot or cold cereals?	◯ Never ◯ 1 time last month<br>◯ 2-3 times last month<br>◯ 1 time per week ◯ 2 times per week<br>◯ 3-4 times per week ◯ 5-6 times per week ◯ 1 time per day<br>◯ 2 or more times per day
During the past month, what kind of cereal did you usually eat?	__________________________________
If there was another kind of cereal that you usually ate during the past month, what kind was it?	__________________________________<br>(Skip if no other kind of cereal)
During the past month (30 days), how often did you drink regular soda or pop that contains sugar? Do not include diet soda.	◯ Never ◯ 1 time last month<br>◯ 2-3 times last month<br>◯ 1 time per week ◯ 2 times per week<br>◯ 3-4 times per week ◯ 5-6 times per week ◯ 1 time per day<br>◯ 2-3 times per day ◯ 4-5 times per day<br>◯ 6 or more times per day
During the past month (30 days), how often did you drink 100% pure fruit juices such as orange, mango, apple, grape and pineapple juices?<br><br>Do not include fruit-flavored drinks with added sugar or fruit juice you made at home and added sugar to.	◯ Never ◯ 1 time last month<br>◯ 2-3 times last month<br>◯ 1 time per week ◯ 2 times per week<br>◯ 3-4 times per week ◯ 5-6 times per week ◯ 1 time per day<br>◯ 2-3 times per day ◯ 4-5 times per day<br>◯ 6 or more times per day
During the past month (30 days), how often did you drink coffee or tea that had sugar or honey added to it?<br><br>Include coffee and tea you sweetened yourself and presweetened tea and coffee drinks such as Arizona Iced Tea and Frappuccino. Do not include artificially sweetened coffee or diet tea.	◯ Never ◯ 1 time last month<br>◯ 2-3 times last month<br>◯ 1 time per week ◯ 2 times per week<br>◯ 3-4 times per week ◯ 5-6 times per week ◯ 1 time per day<br>◯ 2-3 times per day ◯ 4-5 times per day<br>◯ 6 or more times per day
During the past month (30 days), how often did you drink sweetened fruit drinks, sports or energy drinks, such as Kool-Aid, lemonade, Hi-C, cranberry drink, Gatorade, Red Bull or Vitamin Water?<br><br>Include fruit juices you made at home and added sugar to. Do not include diet drinks or artificially sweetened drinks.	◯ Never ◯ 1 time last month<br>◯ 2-3 times last month<br>◯ 1 time per week ◯ 2 times per week<br>◯ 3-4 times per week ◯ 5-6 times per week ◯ 1 time per day<br>◯ 2-3 times per day ◯ 4-5 times per day<br>◯ 6 or more times per day

During the past month (30 days), how often did you eat fruit?<br><br>Include fresh, frozen, or canned fruit. Do not include juices.	◯ Never ◯ 1 time last month<br>◯ 2-3 times last month<br>◯ 1 time per week ◯ 2 times per week<br>◯ 3-4 times per week ◯ 5-6 times per week ◯ 1 time per day<br>◯ 2 or more times per day
During the past month (30 days), how often did you eat a green leafy or lettuce salad, with or without other vegetables?	◯ Never ◯ 1 time last month<br>◯ 2-3 times last month<br>◯ 1 time per week ◯ 2 times per week<br>◯ 3-4 times per week ◯ 5-6 times per week ◯ 1 time per day<br>◯ 2 or more times per day
During the past month (30 days), how often did you eat any kind of fried potatoes, including French fries, home fries, or hash brown potatoes?	◯ Never ◯ 1 time last month<br>◯ 2-3 times last month<br>◯ 1 time per week ◯ 2 times per week<br>◯ 3-4 times per week ◯ 5-6 times per week ◯ 1 time per day<br>◯ 2 or more times per day
During the past month (30 days), how often did you eat any other kind of potatoes, such as baked, boiled, mashed potatoes, sweet potatoes, or potato salad?	◯ Never ◯ 1 time last month<br>◯ 2-3 times last month<br>◯ 1 time per week ◯ 2 times per week<br>◯ 3-4 times per week ◯ 5-6 times per week ◯ 1 time per day<br>◯ 2 or more times per day
During the past month (30 days), how often did you eat refried beans, baked beans, beans in soup, pork and beans or any other type of cooked dried beans?<br><br>Do not include green beans.	◯ Never<br>◯ 1 time last month<br>◯ 2-3 times last month<br>◯ 1 time per week<br>◯ 2 times per week<br>◯ 3-4 times per week<br>◯ 5-6 times per week<br>◯ 1 time per day<br>◯ 2 or more times per day
During the past month (30 days), how often did you eat brown rice or other cooked whole grains, such as bulgur, cracked wheat, or millet? Do not include white rice.	◯ Never ◯ 1 time last month<br>◯ 2-3 times last month<br>◯ 1 time per week ◯ 2 times per week<br>◯ 3-4 times per week ◯ 5-6 times per week ◯ 1 time per day<br>◯ 2 or more times per day
During the past month (30 days), not including the foods you just reported on (i.e., green salads, potatoes, cooked dried beans), how often did you eat other vegetables?	◯ Never ◯ 1 time last month<br>◯ 2-3 times last month<br>◯ 1 time per week ◯ 2 times per week<br>◯ 3-4 times per week ◯ 5-6 times per week ◯ 1 time per day<br>◯ 2 or more times per day

During the past month, how often did you have Mexican-type salsa made with tomato?	◯ Never ◯ 1 time last month ◯ 2-3 times last month ◯ 1 time per week ◯ 2 times per week ◯ 3-4 times per week ◯ 5-6 times per week ◯ 1 time per day ◯ 2 or more times per day
During the past month (30 days), how often did you eat pizza? Include frozen pizza, fast food pizza, and homemade pizza.	◯ Never ◯ 1 time last month ◯ 2-3 times last month ◯ 1 time per week ◯ 2 times per week ◯ 3-4 times per week ◯ 5-6 times per week ◯ 1 time per day ◯ 2 or more times per day
During the past month (30 days), how often did you have tomato sauces such as with spaghetti or noodles or mixed into foods such as lasagna? Do not include tomato sauce on pizza.	◯ Never ◯ 1 time last month ◯ 2-3 times last month ◯ 1 time per week ◯ 2 times per week ◯ 3-4 times per week ◯ 5-6 times per week ◯ 1 time per day ◯ 2 or more times per day
During the past month (30 days), how often did you eat whole grain bread including toast, rolls and in sandwiches? Whole grain breads include whole wheat, rye, oatmeal and pumpernickel. Do not include white bread.	◯ Never ◯ 1 time last month ◯ 2-3 times last month ◯ 1 time per week ◯ 2 times per week ◯ 3-4 times per week ◯ 5-6 times per week ◯ 1 time per day ◯ 2 or more times per day
During the past month (30 days), how often did you eat chocolate or any other types of candy? Do not include sugar-free candy.	◯ Never ◯ 1 time last month ◯ 2-3 times last month ◯ 1 time per week ◯ 2 times per week ◯ 3-4 times per week ◯ 5-6 times per week ◯ 1 time per day ◯ 2 or more times per day
During the past month (30 days), how often did you eat doughnuts, sweet rolls, Danish, muffins, pan dulce, or pop-tarts? Do not include sugar-free items.	◯ Never ◯ 1 time last month ◯ 2-3 times last month ◯ 1 time per week ◯ 2 times per week ◯ 3-4 times per week ◯ 5-6 times per week ◯ 1 time per day ◯ 2 or more times per day
During the past month (30 days), how often did you eat cookies, cake, pie, or brownies? Do not include sugar-free kinds.	◯ Never ◯ 1 time last month ◯ 2-3 times last month ◯ 1 time per week ◯ 2 times per week ◯ 3-4 times per week ◯ 5-6 times per week ◯ 1 time per day ◯ 2 or more times per day

During the past month (30 days), how often did you eat ice cream or other frozen desserts? Do not include sugar-free kinds.	◯ Never ◯ 1 time last month ◯ 2-3 times last month ◯ 1 time per week ◯ 2 times per week ◯ 3-4 times per week ◯ 5-6 times per week ◯ 1 time per day ◯ 2-3 times per day ◯ 4-5 times per day ◯ 6 or more times per day
During the past month (30 days), how often did you eat popcorn?	◯ Never ◯ 1 time last month ◯ 2-3 times last month ◯ 1 time per week ◯ 2 times per week ◯ 3-4 times per week ◯ 5-6 times per week ◯ 1 time per day ◯ 2-3 times per day ◯ 4-5 times per day ◯ 6 or more times per day

Part F. Household Spending

About how much did your household spend in the last month (30 days) on the following? Enter $0 if you did not spend any money in that category

a. Mortgage (interest and principal) ______________________

b. Rent ______________________

c. Electricity ______________________

d. Water ______________________

e. Heating fuel for the house ______________________

f. Cable, internet and land line phone ______________________

g. Cell phone ______________________

Transportation

About how much did your household spend in the last month (30 days) on the following?

Enter $0 if you did not spend any money in that category

a. Car or vehicle payments (interest and principal) __________________________________

b. Car or vehicle insurance __________________________________

c. Gasoline __________________________________

d. Public transit __________________________________

e. Other transportation expenses (parking, tolls, taxi, and rideshares apps like Uber) __________________________________

Healthcare spending

About how much did your household spend in the last month (30 days) on the following?

Enter $0 if you did not spend any money in that category

a. Health insurance premium ______________________

b. Prescription and nonprescription medications (Include only out-of-pocket expenses. Do not include what is covered by insurance) ______________________

c. Nursing home care or at-home care service (Include only out-of-pocket expenses. Do not include what is covered by insurance) ______________________

d. Eye care (Include only out-of-pocket expenses. Do not include what is covered by insurance) ______________________

e. Dental care (Include only out-of-pocket expenses. Do not include what is covered by insurance) ______________________

f. Other medical expenses, such as copays (Include only out-of-pocket expenses. Do not include what is covered by insurance) ______________________

Other spending or saving

About how much did your household spend or save in the last month (30 days) on the following?

Enter $0 if you did not spend or save any money in that category

a. Exercise/sports (including gym, exercise equipment such as bicycles, skis, etc.) ______________________

b. Childcare (including daycare, babysitters, and afterschool care) ______________________

c. Cash or gifts to family or friends outside the household ______________________

d. Savings ______________________

Did you purchase any of the following in the last year (12 months)?

Include newly purchased items not fully paid for at the time of purchase.

Check all that apply

- ☐ Automobile or truck
- ☐ Major vehicle repair
- ☐ Large home appliance (refrigerator, stove/oven, dishwasher, washing machine, dryer, etc.)
- ☐ Major home repair (furnace, roof, walls, etc.)
- ☐ Major home furniture (couch/sofa, bed, etc.)
- ☐ Television
- ☐ Computer
- ☐ Trip or vacation
- ☐ None of the above

Spending Reconciliation

Below is a summary of your household spending entries. Please review your entries and make adjustments as needed.

Mortgage= __________________________________

Rent= __________________________________

Electricity= __________________________________

Water= __________________________________

Heating= __________________________________

Cable/Internet/Land line phone= __________________________________

Cell phone= __________________________________

Car payment= __________________________________

Car insurance= __________________________________

Gasoline= __________________________________

Public transit= __________________________________

Other transportation= __________________________________

Health insurance= __________________________________

Medications= __________________________________

Nursing home/at-home care= __________________________________

Eye care= __________________________________

Dental care= __________________________________

Exercise= __________________________________

Childcare= __________________________________

Cash/gifts to others= __________________________________

Savings= __________________________________

2019 (T2) Participant Survey_English

Please complete the survey below.

Thank you!

How many adults (age 18 or older) currently live in your household (including yourself)?	◯ 1<br>◯ 2<br>◯ 3<br>◯ 4<br>◯ 5 or more
How many children (under the age of 18) currently live in your household, including children who live there part-time?	◯ 0<br>◯ 1<br>◯ 2<br>◯ 3<br>◯ 4<br>◯ 5 or more

Please list the following information for each child living in the household:

Child 1 Age

(Enter months if under age 1)

Years ______________________

Months ______________________

Child 1 Weight ______________________
(Pounds (lbs))

Child 1 Height ______________________
(Inches)

Child 2 Age

(Enter months if under age 1)

Years ______________________

Months ______________________

Child 2 Weight ______________________
(Pounds (lbs))

Child 2 Height ______________________
(Inches)

Child 3 Age

(enter months if under age 1)

Years ______________________

Months ______________________

Child 3 Weight ______________________
(Pounds (lbs))

Child 3 Height ______________________
(Inches)

Child 4 Age

(Enter months if under age 1)

Years ______________________

Months ______________________

Child 4 Weight ______________________
(Pounds (lbs))

Child 4 Height ______________________
(Inches)

Child 5 Age

(enter months if under age 1)

Years ______________________

Months ______________________

Child 5 Weight ______________________
(Pounds (lbs))

Child 5 Height ______________________
(Inches)

How did you determine the child or children's height or weight?	◯ Pediatrician or health care provider records<br>◯ School records<br>◯ Measured at home<br>◯ My best guess<br>◯ Other
If you selected other, please specify:	__________________________
Are you registered to vote?	◯ Yes<br>◯ No
Did you vote in the last local election, like for mayor or city council member?	◯ Yes<br>◯ No
Did you vote in the last general election, like for state representatives, senators and president?	◯ Yes<br>◯ No
In the last year (12 months), was there a time when you were not able to pay the mortgage or rent on time?	◯ Yes<br>◯ No
In the last year (12 months), how many places have you lived?	◯ 0<br>◯ 1<br>◯ 2<br>◯ 3<br>◯ More than 3
In the last year (12 months), was there a time when you did not have a steady place to sleep or slept in a shelter (including now)?	◯ Yes<br>◯ No
What is the highest grade or level of school you have completed?	◯ Less than High School<br>◯ Some High School<br>◯ High School Diploma<br>◯ Associate/Technical Degree<br>◯ Some College<br>◯ Bachelor's Degree<br>◯ Graduate Degree
When did you complete this schooling?	____________________<br>(Year (YYYY))
Have you completed any of the following types of trainings/career development activities during the last year (12 months)?<br>(Check all that apply)	☐ A training, workshop, seminar or professional event required by your employer<br>☐ A training, workshop, seminar or professional event not required by your employer<br>☐ Licensure or certification for your occupation<br>☐ A training, workshop, seminar or professional development event to help you change to a new occupation<br>☐ Other<br>☐ None of the above

If you selected other, please specify:	__________________________
What was your annual household income (from all sources) last year, before taxes?	◯ Less than $5,000<br>◯ $5,001 to $10,000<br>◯ $10,001 to $20,000<br>◯ $20,001 to $30,000<br>◯ $30,001 to $40,000<br>◯ $40,001 to $50,000<br>◯ More than $50,000
Do you own or have access to a vehicle for reliable transportation?	◯ Yes<br>◯ No

In the last month (30 days) did you or anyone in your household receive any of the following? (Check Yes or No for EACH program)

	Yes	No	Not Sure
WIC (Women, Infants, and Children program)	◯	◯	◯
Food stamps (SNAP)	◯	◯	◯
Free or reduced price school lunch	◯	◯	◯
Minnesota Family Investment Program (You may recieve one or more of the following together or seperate: Cash, Food support, childcare assistance, housing grant.)	◯	◯	◯
State housing subsidy (e.g., Bridges Housing Subsidy, HUD Rental Assistance, Housing Choice/Housing Choice vouchers, Public Housing, Section 42 Housing)	◯	◯	◯
Disability assistance (including VA disability assistance)	◯	◯	◯
Work First (Temporary Assistance for the Needy Families, TANF)	◯	◯	◯

How much did your household receive in SNAP benefits in the last month (30 days)?

- ◯ I do not receive any food stamps or SNAP benefits
- ◯ $1 - $25
- ◯ $26 - $50
- ◯ $51 - $75
- ◯ $76 - $100
- ◯ $101 - $150
- ◯ $151 - $250
- ◯ $251 - $500
- ◯ $501 - $750
- ◯ More than $750

Did your wages (dollars per hour) increase in the last 12 months?

- ◯ Yes
- ◯ No

If Yes, has your increase in wages made you worry about losing any public assistance? (housing subsidy, SNAP, WIC, MFIP, healthcare assistance)

- ◯ Yes
- ◯ No

Has your worry about losing assistance caused you to make job related changes? (quit job, asked to have hours reduced, got a different job)

- ◯ Yes
- ◯ No

Please explain the job changes you made because you were worried about losing public assistance.

____

Have you lost any public assistance in the last 12 months?	◯ Yes<br>◯ No
What public assistance did you lose in the last 12 months?	☐ WIC (Women, Infants, and Children program)<br>☐ Food stamps (SNAP)<br>☐ Free or reduced price school lunch<br>☐ Minnesota Family Investment Program (MFIP: Cash, Food support, childcare assistance, housing grant.)<br>☐ State housing subsidy (e.g., Bridges Housing Subsidy, HUD Rental Assistance, Housing Choice/Housing Choice vouchers, Public Housing, Section 42 Housing))<br>☐ Healthcare assistance<br>☐ Disability Assistance (including VA disability assistance)<br>☐ Work First (TANF)<br>☐ Other
Please explain the other public assistance you lost.	__________________________________
What happened that caused you to lose this assistance?	__________________________________

Part B. Work and Finances

What is your average monthly income from wages (i.e. pay for the jobs you yourself work)?	◯ Less than $500<br>◯ $501 to $1,000<br>◯ $1,001 to $1,500<br>◯ $1,501 to $2,000<br>◯ $2,001 to $2,500<br>◯ More than $2,500
Do you have a physical health condition or disability that affects the number of hours of work you perform in a week?	◯ Yes<br>◯ No
Do you have mental health condition or disability that affects the number of hours of work you perform in a week?	◯ Yes<br>◯ No
Which one of the following best describes how well you are managing with money these days:	◯ Living comfortably<br>◯ Doing okay<br>◯ Just getting by<br>◯ Finding it difficult to get by
Compared to one year (12 months) ago, would you say that you (and your family living with you) are better off, the same, or worse off with money?	◯ Much better off<br>◯ Somewhat better off<br>◯ About the same<br>◯ Somewhat worse off<br>◯ Much worse off
Which of the following hardships did you or your family experience in the last year (12 months)? (Check all that apply)	☐ I lost a job<br>☐ I had my hours reduced<br>☐ I changed jobs<br>☐ I was suspended from work without pay<br>☐ I had a medical procedure or health emergency<br>☐ I experienced the death of another income earner<br>☐ I began caring for a loved one<br>☐ I was incarcerated<br>☐ I was arrested/charged with a crime<br>☐ I experienced divorce/separation<br>☐ I experienced the birth of a child<br>☐ I and/or my family lost transportation (vehicle repossessed or totaled, bus service cut etc...)<br>☐ I and/or my family experienced homelessness (homeless or displaced from home)<br>☐ I and/or my family received a foreclosure or eviction<br>☐ I and/or my family experienced the death of a loved one<br>☐ My partner/spouse lost their job<br>☐ My partner/spouse had their hours reduced<br>☐ Other<br>☐ None of the above
If you selected other, please specify:	__________________________________

During the past twelve months, did you (or your {husband/wife/partner}) do any of the following? (check all that apply)

- ☐ Apply for a credit card or respond to a pre-approved credit card offer
- ☐ Request an increase in the credit limit of a credit card
- ☐ Apply for a mortgage or home-based loan
- ☐ Request to refinance a mortgage
- ☐ Apply for an auto loan
- ☐ Apply for a student loan
- ☐ Request an increase in the limit of an existing loan (other than a credit card)

How do you pay your bills? (Check all that apply)

- ☐ Electronic payment from bank
- ☐ Personal check
- ☐ Debit card
- ☐ Credit card
- ☐ Bank money order
- ☐ Cash
- ☐ Non-bank money order
- ☐ Pre-paid card
- ☐ Other

What other method do you use to pay your bills?

____

Have you used any of the following services in the last year (12 months)? (Check all that apply)

- ☐ Pawn Shop
- ☐ Pay Day Loan
- ☐ Auto Title Loan
- ☐ Paycheck Advance Service
- ☐ Tax Refund Anticipation Loan
- ☐ Borrowed money from a friend, family or community member
- ☐ Got a loan from my employer
- ☐ None of the above

If you were faced with a $400 emergency expense today, how would you most likely cover it?

(Check all that apply)

- ☐ Use money from recent paycheck or savings
- ☐ Borrow money from a friend, family or community member
- ☐ Put it on my credit card and pay it off over time
- ☐ Sell something you own
- ☐ Delay payment on other bills or expenses
- ☐ Pawn Shop
- ☐ Pay Day Loan or Paycheck Advance Service
- ☐ Auto Title Loan
- ☐ Tax Refund Anticipation Loan
- ☐ Ask my employer for a loan
- ☐ I do not know how I would cover this expense
- ☐ Other

If you were faced with a $500 emergency expense today, how would you most likely cover it?<br><br>(Check all that apply)	☐ Use money from recent paycheck or savings<br>☐ Borrow money from a friend, family or community member<br>☐ Put it on my credit card and pay it off over time<br>☐ Sell something you own<br>☐ Delay payment on other bills or expenses<br>☐ Pawn Shop<br>☐ Pay Day Loan or Paycheck Advance Service<br>☐ Auto Title Loan<br>☐ Tax Refund Anticipation Loan<br>☐ Ask my employer for a loan<br>☐ I do not know how I would cover this expense<br>☐ Other
If you were faced with a $600 emergency expense today, how would you most likely cover it?<br><br>(Check all that apply)	☐ Use money from recent paycheck or savings<br>☐ Borrow money from a friend, family or community member<br>☐ Put it on my credit card and pay it off over time<br>☐ Sell something you own<br>☐ Delay payment on other bills or expenses<br>☐ Pawn Shop<br>☐ Pay Day Loan or Paycheck Advance Service<br>☐ Auto Title Loan<br>☐ Tax Refund Anticipation Loan<br>☐ Ask my employer for a loan<br>☐ I do not know how I would cover this expense<br>☐ Other
In the past year, have you put off paying a bill because you didn't have enough money to pay it when it was due?	○ Yes<br>○ No
Do you have an account with a bank or credit union?	○ Yes<br>○ No

Part C. Accessing Food

What is the name and location of the store where MOST of the food for your household is purchased?

Store/Location Name: ______________________

Store/Location address (or nearby streets/cross streets) ______________________

Store/Location City: ______________________

Who usually shops for most of the food for your household?

(Choose more than one if the task is evenly split)

- ☐ Me
- ☐ Spouse/Partner
- ☐ Child/Children
- ☐ Other adult in the home
- ☐ Other

If you selected other, please specify: ______________________

How do you usually get to the store where you get most of your food?

- ◯ Drive myself in a vehicle
- ◯ Family member/friends drive me in their vehicle
- ◯ Take public transportation (e.g. bus)
- ◯ Walk
- ◯ Taxi, Uber, or other car service
- ◯ Bicycle
- ◯ Other

If you selected other, please specify: ______________________

Please indicate how often each statement below was true for your household in the last year (12 months):

	Often true	Sometimes true	Never true
The food that we bought just didn't last and we didn't have money to get more.	◯	◯	◯
We couldn't afford to eat balanced meals.	◯	◯	◯

In the last year (12 months), did you or other adults in the household ever cut the size of your meal or skip meals because there was not enough money for food?

◯ Yes
◯ No
◯ Don't Know

How often did this happen?

◯ Almost every month
◯ Some months but not every month
◯ 1 or 2 months

In the last year (12 months), did you ever eat less than you felt you should because there wasn't enough money for food?

◯ Yes
◯ No
◯ Don't Know

In the last year (12 months), were you ever hungry but didn't eat because there wasn't enough money for food?

◯ Yes
◯ No
◯ Don't Know

Part D. Your Health

In general, would you say your health is:	◯ Excellent<br>◯ Very good<br>◯ Good<br>◯ Fair<br>◯ Poor
Are you currently pregnant?	◯ Yes<br>◯ No
What type(s) of health insurance/health coverage do you currently have?<br><br>(Check all that apply)	☐ Medicaid<br>☐ Medicare<br>☐ Health insurance plan offered through your employer<br>☐ Health insurance through your parent's insurance plan/employer<br>☐ Health insurance plan obtained through Healthcare.gov or MNsure (ACA Exchange)<br>☐ Private Insurance Plan (not obtained through insurance exchange/MNSure)<br>☐ Veterans' Administration (VA) Health Benefits<br>☐ Indian Health Services (IHS) tribal and/or urban Indian health programs<br>☐ Uninsured<br>☐ Other
If you selected other, please specify:	______________________________
When was the last time you visited a doctor, physician assistant or nurse in a clinic or medical office?<br><br>Do not include times you were hospitalized overnight or visits to the hospital emergency room.	◯ Within the last 12 months<br>◯ Within the last 1-2 years<br>◯ Within the last 2-5 years<br>◯ More than 5 years ago<br>◯ Never
In the last year (12 months), did you have an illness, injury, or condition that needed care right away in a clinic, emergency room, or doctor's office?	◯ Yes<br>◯ No
In the last year (12 months), when you needed care right away, how often did you get care as soon as you thought you needed it?	◯ Never<br>◯ Sometimes<br>◯ Usually<br>◯ Always
In the last year (12 months), not counting the times you needed care right away, did you make appointments for your health care at a doctor's office or clinic?	◯ Yes<br>◯ No
In the last year (12 months), how often did doctors or other health providers listen carefully to you?	◯ Never<br>◯ Sometimes<br>◯ Usually<br>◯ Always
In the last year (12 months), how often did doctors or other health providers show respect for what you had to say?	◯ Never<br>◯ Sometimes<br>◯ Usually<br>◯ Always

In the last year (12 months), how often did doctors or other health providers spend enough time with you?

◯ Never
◯ Sometimes
◯ Usually
◯ Always

Please check one of the boxes to indicate how strongly you agree or disagree for each statement.

	Strongly Disagree	Somewhat Disagree	Neutral	Somewhat Agree	Strongly Agree
I'm healthy enough that I really don't need health insurance.	○	○	○	○	○
Health insurance is not worth the money it costs.	○	○	○	○	○
I'm more likely to take risks than the average person.	○	○	○	○	○
I can overcome illness without help from a medically trained person.	○	○	○	○	○

Considering a 7-Day period (a week), how many times on the average do you do the following kinds of exercise for more than 15 minutes during your free time:

STRENUOUS EXERCISE
(Heart beats rapidly)
Examples: running, jogging, hockey, football, soccer, basketball, cross-country skiing, roller blading, vigorous swimming, vigorous bicycling

____
(_____ times/week)

MODERATE EXERCISE
(Not exhausting)
Examples: fast walking, baseball, tennis, easy bicycling, badminton, easy swimming, downhill skiing, recreational dancing

____
(_____ times/week)

MILD EXERCISE
(Minimal effort)
Examples: easy walking, yoga, fishing, bowling, golf

____
(_____ times/week)

How much time do you usually spend sitting or reclining on a typical day?

Include time spent sitting at work, at home, getting to and from places (i.e. in a car, bus or train).
Do not include time spent sleeping.

____
(Hours)

How much time did you spend last week playing video games?

____
(Hours)

What time do you usually go to bed (turn off the lights to sleep)?

◯ 1 AM ◯ 2 AM ◯ 3 AM
◯ 4 AM ◯ 5 AM ◯ 6 AM
◯ 7 AM ◯ 8 AM ◯ 9 AM
◯ 10 AM ◯ 11 AM ◯ 12 PM (noon)
◯ 1 PM ◯ 2 PM ◯ 3 PM
◯ 4 PM ◯ 5 PM ◯ 6 PM
◯ 7 PM ◯ 8 PM ◯ 9 PM
◯ 10 PM ◯ 11 PM ◯ 12 AM (midnight)

What time do you usually get out of bed?	◯ 1 AM ◯ 2 AM ◯ 3 AM<br>◯ 4 AM ◯ 5 AM ◯ 6 AM<br>◯ 7 AM ◯ 8 AM ◯ 9 AM<br>◯ 10 AM ◯ 11 AM ◯ 12 PM (noon)<br>◯ 1 PM ◯ 2 PM ◯ 3 PM<br>◯ 4 PM ◯ 5 PM ◯ 6 PM<br>◯ 7 PM ◯ 8 PM ◯ 9 PM<br>◯ 10 PM ◯ 11 PM ◯ 12 AM (midnight)
Please select one of the following:	◯ Current smoker ◯ Quit less than 12 months ago ◯ Quit more than 12 months ago ◯ Never smoked

What product(s) do you smoke most regularly?
(Check all that apply)

☐ Cigarettes
☐ Cigars, cigarillos, or little cigars
☐ E-cigarettes (Juul, e-cig, vaping pen)
☐ Tobacco in a hookah or waterpipe
☐ Pipes filled with tobacco (not waterpipes)
☐ Bidis (small brown cigarettes wrapped in a leaf)
☐ Marijuana
☐ Other

Please indicate how often each statement below was true for you in the last month (30 days)

	Never	Almost Never	Sometimes	Fairly Often	Very Often
a. In the last month (30 days), how often have you felt unable to control the important things in your life?	○	○	○	○	○
b. In the last month (30 days), how often have you felt confident about your ability to handle your personal problems?	○	○	○	○	○
c. In the last month (30 days), how often have you felt that things were going your way?	○	○	○	○	○
d. In the last month (30 days), how often have you felt difficulties were piling up so high that you could not overcome them?	○	○	○	○	○

Part E. Dietary Screener Questionnaire

These questions are about foods you ate or drank during the past month, that is, the past 30 days. When answering, please include meals and snacks at home, at work or school, in restaurants and anyplace else.

During the past month (30 days), how often did you eat hot or cold cereals?	◯ Never ◯ 1 time last month ◯ 2-3 times last month ◯ 1 time per week ◯ 2 times per week ◯ 3-4 times per week ◯ 5-6 times per week ◯ 1 time per day ◯ 2 or more times per day
During the past month, what kind of cereal did you usually eat?	______________________________
If there was another kind of cereal that you usually ate during the past month, what kind was it?	______________________________ (Skip if no other kind of cereal)
During the past month (30 days), how often did you drink regular soda or pop that contains sugar? Do not include diet soda.	◯ Never ◯ 1 time last month ◯ 2-3 times last month ◯ 1 time per week ◯ 2 times per week ◯ 3-4 times per week ◯ 5-6 times per week ◯ 1 time per day ◯ 2-3 times per day ◯ 4-5 times per day ◯ 6 or more times per day
During the past month (30 days), how often did you drink 100% pure fruit juices such as orange, mango, apple, grape and pineapple juices?<br><br>Do not include fruit-flavored drinks with added sugar or fruit juice you made at home and added sugar to.	◯ Never ◯ 1 time last month ◯ 2-3 times last month ◯ 1 time per week ◯ 2 times per week ◯ 3-4 times per week ◯ 5-6 times per week ◯ 1 time per day ◯ 2-3 times per day ◯ 4-5 times per day ◯ 6 or more times per day
During the past month (30 days), how often did you drink coffee or tea that had sugar or honey added to it?<br><br>Include coffee and tea you sweetened yourself and presweetened tea and coffee drinks such as Arizona Iced Tea and Frappuccino. Do not include artificially sweetened coffee or diet tea.	◯ Never ◯ 1 time last month ◯ 2-3 times last month ◯ 1 time per week ◯ 2 times per week ◯ 3-4 times per week ◯ 5-6 times per week ◯ 1 time per day ◯ 2-3 times per day ◯ 4-5 times per day ◯ 6 or more times per day
During the past month (30 days), how often did you drink sweetened fruit drinks, sports or energy drinks, such as Kool-Aid, lemonade, Hi-C, cranberry drink, Gatorade, Red Bull or Vitamin Water?<br><br>Include fruit juices you made at home and added sugar to. Do not include diet drinks or artificially sweetened drinks.	◯ Never ◯ 1 time last month ◯ 2-3 times last month ◯ 1 time per week ◯ 2 times per week ◯ 3-4 times per week ◯ 5-6 times per week ◯ 1 time per day ◯ 2-3 times per day ◯ 4-5 times per day ◯ 6 or more times per day

During the past month (30 days), how often did you eat fruit?<br><br>Include fresh, frozen, or canned fruit. Do not include juices.	◯ Never ◯ 1 time last month<br>◯ 2-3 times last month<br>◯ 1 time per week ◯ 2 times per week<br>◯ 3-4 times per week ◯ 5-6 times per week ◯ 1 time per day<br>◯ 2 or more times per day
During the past month (30 days), how often did you eat a green leafy or lettuce salad, with or without other vegetables?	◯ Never ◯ 1 time last month<br>◯ 2-3 times last month<br>◯ 1 time per week ◯ 2 times per week<br>◯ 3-4 times per week ◯ 5-6 times per week ◯ 1 time per day<br>◯ 2 or more times per day
During the past month (30 days), how often did you eat any kind of fried potatoes, including French fries, home fries, or hash brown potatoes?	◯ Never ◯ 1 time last month<br>◯ 2-3 times last month<br>◯ 1 time per week ◯ 2 times per week<br>◯ 3-4 times per week ◯ 5-6 times per week ◯ 1 time per day<br>◯ 2 or more times per day
During the past month (30 days), how often did you eat any other kind of potatoes, such as baked, boiled, mashed potatoes, sweet potatoes, or potato salad?	◯ Never ◯ 1 time last month<br>◯ 2-3 times last month<br>◯ 1 time per week ◯ 2 times per week<br>◯ 3-4 times per week ◯ 5-6 times per week ◯ 1 time per day<br>◯ 2 or more times per day
During the past month (30 days), how often did you eat refried beans, baked beans, beans in soup, pork and beans or any other type of cooked dried beans?<br><br>Do not include green beans.	◯ Never<br>◯ 1 time last month<br>◯ 2-3 times last month<br>◯ 1 time per week<br>◯ 2 times per week<br>◯ 3-4 times per week<br>◯ 5-6 times per week<br>◯ 1 time per day<br>◯ 2 or more times per day
During the past month (30 days), how often did you eat brown rice or other cooked whole grains, such as bulgur, cracked wheat, or millet? Do not include white rice.	◯ Never ◯ 1 time last month<br>◯ 2-3 times last month<br>◯ 1 time per week ◯ 2 times per week<br>◯ 3-4 times per week ◯ 5-6 times per week ◯ 1 time per day<br>◯ 2 or more times per day
During the past month (30 days), not including the foods you just reported on (i.e., green salads, potatoes, cooked dried beans), how often did you eat other vegetables?	◯ Never ◯ 1 time last month<br>◯ 2-3 times last month<br>◯ 1 time per week ◯ 2 times per week<br>◯ 3-4 times per week ◯ 5-6 times per week ◯ 1 time per day<br>◯ 2 or more times per day

During the past month, how often did you have Mexican-type salsa made with tomato?	◯ Never ◯ 1 time last month<br>◯ 2-3 times last month<br>◯ 1 time per week ◯ 2 times per week<br>◯ 3-4 times per week ◯ 5-6 times per week ◯ 1 time per day<br>◯ 2 or more times per day
During the past month (30 days), how often did you eat pizza? Include frozen pizza, fast food pizza, and homemade pizza.	◯ Never ◯ 1 time last month<br>◯ 2-3 times last month<br>◯ 1 time per week ◯ 2 times per week<br>◯ 3-4 times per week ◯ 5-6 times per week ◯ 1 time per day<br>◯ 2 or more times per day
During the past month (30 days), how often did you have tomato sauces such as with spaghetti or noodles or mixed into foods such as lasagna? Do not include tomato sauce on pizza.	◯ Never ◯ 1 time last month<br>◯ 2-3 times last month<br>◯ 1 time per week ◯ 2 times per week<br>◯ 3-4 times per week ◯ 5-6 times per week ◯ 1 time per day<br>◯ 2 or more times per day
During the past month (30 days), how often did you eat whole grain bread including toast, rolls and in sandwiches? Whole grain breads include whole wheat, rye, oatmeal and pumpernickel. Do not include white bread.	◯ Never ◯ 1 time last month<br>◯ 2-3 times last month<br>◯ 1 time per week ◯ 2 times per week<br>◯ 3-4 times per week ◯ 5-6 times per week ◯ 1 time per day<br>◯ 2 or more times per day
During the past month (30 days), how often did you eat chocolate or any other types of candy? Do not include sugar-free candy.	◯ Never ◯ 1 time last month<br>◯ 2-3 times last month<br>◯ 1 time per week ◯ 2 times per week<br>◯ 3-4 times per week ◯ 5-6 times per week ◯ 1 time per day<br>◯ 2 or more times per day
During the past month (30 days), how often did you eat doughnuts, sweet rolls, Danish, muffins, pan dulce, or pop-tarts? Do not include sugar-free items.	◯ Never ◯ 1 time last month<br>◯ 2-3 times last month<br>◯ 1 time per week ◯ 2 times per week<br>◯ 3-4 times per week ◯ 5-6 times per week ◯ 1 time per day<br>◯ 2 or more times per day
During the past month (30 days), how often did you eat cookies, cake, pie, or brownies? Do not include sugar-free kinds.	◯ Never ◯ 1 time last month<br>◯ 2-3 times last month<br>◯ 1 time per week ◯ 2 times per week<br>◯ 3-4 times per week ◯ 5-6 times per week ◯ 1 time per day<br>◯ 2 or more times per day

During the past month (30 days), how often did you eat ice cream or other frozen desserts? Do not include sugar-free kinds.	◯ Never ◯ 1 time last month<br>◯ 2-3 times last month<br>◯ 1 time per week ◯ 2 times per week<br>◯ 3-4 times per week ◯ 5-6 times per week ◯ 1 time per day<br>◯ 2-3 times per day ◯ 4-5 times per day<br>◯ 6 or more times per day
During the past month (30 days), how often did you eat popcorn?	◯ Never ◯ 1 time last month<br>◯ 2-3 times last month<br>◯ 1 time per week ◯ 2 times per week<br>◯ 3-4 times per week ◯ 5-6 times per week ◯ 1 time per day<br>◯ 2-3 times per day ◯ 4-5 times per day<br>◯ 6 or more times per day

Part F. Household Spending

About how much did your household spend in the last month (30 days) on the following? Enter $0 if you did not spend any money in that category, Enter N/A if this category does not apply to you.

a. Mortgage (including taxes, interest and principal) __________________________________

b. Rent __________________________________

c. Electricity __________________________________

d. Water __________________________________

e. Heating fuel for the house __________________________________

f. Cable, internet and land line phone payment amount __________________________________

g. Cell phone __________________________________

h. Groceries __________________________________

i. Other food (restaurants, take-out) __________________________________

Transportation
About how much did your household spend in the last month (30 days) on the following? Enter $0 if you did not spend any money in that category, Enter N/A if this category does not apply to you.

a. Car or vehicle payments (interest and principal) ________________________________

b. Car or vehicle insurance ________________________________

c. Gasoline ________________________________

d. Public transit ________________________________

e. Other transportation expenses (parking, tolls, taxi, and rideshares apps like Uber) ________________________________

Healthcare spending

About how much did your household spend in the last month (30 days) on the following? Enter $0 if you did not spend any money in that category. Enter N/A if this category does not apply to you.

a. Health insurance premium ______________________

b. Prescription and nonprescription medications (Include only out-of-pocket expenses. Do not include what is covered by insurance) ______________________

c. Nursing home care or at-home care service (Include only out-of-pocket expenses. Do not include what is covered by insurance) ______________________

d. Eye care (Include only out-of-pocket expenses. Do not include what is covered by insurance) ______________________

e. Dental care (Include only out-of-pocket expenses. Do not include what is covered by insurance) ______________________

f. Other medical expenses, such as copays (Include only out-of-pocket expenses. Do not include what is covered by insurance) ______________________

Other spending or saving

About how much did your household spend or save in the last month (30 days) on the following?

Enter $0 if you did not spend or save any money in that category. Enter N/A if this category does not apply to you.

a. Exercise/sports (including gym, exercise equipment such as bicycles, skis, etc.) ______________________

b. Childcare (including daycare, babysitters, and afterschool care) ______________________

c. Cash or gifts to family or friends outside the household (including "tithes" or donations to churches/synagogues/mosques) ______________________

d. Savings ______________________

e. Child support ______________________

f. Student loan payments ______________________

Did you purchase any of the following in the last year (12 months)?

Include newly purchased items not fully paid for at the time of purchase.

Check all that apply

- ☐ Automobile or truck
- ☐ Major vehicle repair
- ☐ Large home appliance (refrigerator, stove/oven, dishwasher, washing machine, dryer, etc.)
- ☐ Major home repair (furnace, roof, walls, etc.)
- ☐ Major home furniture (couch/sofa, bed, etc.)
- ☐ Television
- ☐ Computer
- ☐ Trip or vacation
- ☐ None of the above

Spending Reconciliation

Below is a summary of your household spending entries. Please review your entries and make adjustments as needed.

Mortgage= ______________________________

Rent= ______________________________

Electricity= ______________________________

Water= ______________________________

Heating= ______________________________

Cable/Internet/Land line phone= ______________________________

Cell phone= ______________________________

Groceries= ______________________________

Other Food= ______________________________

Car payment= ______________________________

Car insurance= ______________________________

Gasoline= ______________________________

Public transit= ______________________________

Other transportation= ______________________________

Health insurance= ______________________________

Medications= ______________________________

Nursing home/at-home care= ______________________________

Eye care= __________________________________

Dental care= __________________________________

Exercise= __________________________________

Childcare= __________________________________

Cash/gifts to others= __________________________________

Savings= __________________________________

Child support= __________________________________

Student loan payments= __________________________________

2020 (T3) Wages Participant Survey

Please take this survey.
You may open the survey in your web browser by clicking the link below:
[survey-link]
If the link above does not work, try copying the link below into your web browser:
[survey-url]
This link is unique to you and should not be forwarded to others.

How many adults (age 18 or older) currently live in your household (including yourself)?

- ○ 1
- ○ 2
- ○ 3
- ○ 4
- ○ 5 or more

How many children (under the age of 18) currently live in your household, including children who live there part-time?

- ○ 0
- ○ 1
- ○ 2
- ○ 3
- ○ 4
- ○ 5 or more

Please list the following information for each child living in the household:

Child 1
Age

______________________ (Years)

Child 1 Age

(Enter months if under age 1)

Years ______________________

Months ______________________

Child 1 Weight ______________________ (Pounds (lbs))

Child 1 Height ______________________ (Feet / Inches)

Child 2 Age

(Enter months if under age 1)

Years ______________________

Months ______________________

Child 2
Age

______________________ (Years)

Child 2 Weight ______________________ (Pounds (lbs))

Child 2 Height ______________________ (Feet / Inches)

Child 3
Age

______________________ (Years)

Child 3 Age

(enter months if under age 1)

Years ______________________

Months	______________________
Child 3 Weight	______________________ (Pounds (lbs))
Child 3 Height	______________________ (Feet / Inches)
Child 4<br>Age	______________________ (Years)
Child 4 Age<br><br>(Enter months if under age 1)	
Years	______________________
Months	______________________
Child 4 Weight	______________________ (Pounds (lbs))
Child 4 Height	______________________ (Feet / Inches)
Child 5<br>Age	______________________ (Years)
Child 5 Age<br><br>(enter months if under age 1)	
Years	______________________
Months	______________________
Child 5 Weight	______________________ (Pounds (lbs))
Child 5 Height	______________________ (Feet / Inches)

How did you determine the child or children's height or weight?

◯ Pediatrician or health care provider records
◯ School records
◯ Measured at home
◯ My best guess
◯ Other

If you selected Other, please specify:

____

Are you registered to vote?

◯ Yes
◯ No

Did you vote in the last local election, like for mayor or city council member?

◯ Yes
◯ No

Did you vote in the last general election, like for state representatives, senators and president?

◯ Yes
◯ No

In the last year (12 months), was there a time when you were not able to pay the mortgage or rent on time?

◯ Yes
◯ No

In the last year (12 months), how many places have you lived?

◯ 0
◯ 1
◯ 2
◯ 3
◯ More than 3

In the last year (12 months), was there a time when you did not have a steady place to sleep or slept in a shelter (including now)?

◯ Yes
◯ No

What is the highest grade or level of school you have completed?

◯ Less than High School
◯ Some High School
◯ High School Diploma
◯ Associate/Technical Degree
◯ Some College
◯ Bachelor's Degree
◯ Graduate Degree

When did you complete this schooling?

____
(Year (YYYY))

Have you completed any of the following types of trainings/career development activities in the last year (12 months)?
(Check all that apply)

☐ A training, workshop, seminar or professional event required by your employer
☐ A training, workshop, seminar or professional event not required by your employer
☐ Licensure or certification for your occupation
☐ A training, workshop, seminar or professional development event to help you change to a new occupation
☐ Other
☐ None of the above

If you selected Other, please specify:

____

What was your annual household income before taxes (from all sources), last year?

◯ Less than $5,000
◯ $5,001 to $10,000
◯ $10,001 to $20,000
◯ $20,001 to $30,000
◯ $30,001 to $40,000
◯ $40,001 to $50,000
◯ More than $50,000

Do you own or have access to a vehicle for reliable transportation?

◯ Yes
◯ No

In the last month (30 days) did you or anyone in your household receive any of the following? (Check Yes or No for EACH program)

	Yes	No	Not Sure
WIC (Women, Infants, and Children program)	◯	◯	◯
Food stamps (SNAP)	◯	◯	◯
Free or reduced price school lunch	◯	◯	◯
MFIP (Minnesota Family Investment Program) (You may recieve one or more of the following together or seperate: Cash, Food support, childcare assistance, housing grant.)	◯	◯	◯
State housing subsidy (e.g., Bridges Housing Subsidy, HUD Rental Assistance, Housing Choice/Housing Choice vouchers, Public Housing, Section 42 Housing)	◯	◯	◯
Disability assistance (including VA disability assistance)	◯	◯	◯
Work First (Temporary Assistance for the Needy Families, TANF)	◯	◯	◯

How much did your household receive in SNAP benefits in the last month (30 days)?

◯ I do not receive any food stamps or SNAP benefits
◯ $1 - $25
◯ $26 - $50
◯ $51 - $75
◯ $76 - $100
◯ $101 - $150
◯ $151 - $250
◯ $251 - $500
◯ $501 - $750
◯ More than $750

Did your wages (dollars per hour) increase in the last year (12 months)?

◯ Yes
◯ No

If Yes, has your increase in wages made you worry about losing any public assistance? (housing subsidy, SNAP, WIC, MFIP, healthcare assistance)

◯ Yes
◯ No

Has your worry about losing assistance caused you to make job related changes? (quit job, asked to have hours reduced, got a different job)

◯ Yes
◯ No

Please explain the job changes you made because you were worried about losing public assistance.

____

Have you lost any public assistance in the last year (12 months)?

◯ Yes
◯ No

What public assistance did you lose in the last year (12 months)?

☐ WIC (Women, Infants, and Children program)
☐ Food stamps (SNAP)
☐ Free or reduced price school lunch
☐ MFIP (Minnesota Family Investment Program): Cash, Food support, childcare assistance, housing grant.)
☐ State housing subsidy (e.g., Bridges Housing Subsidy, HUD Rental Assistance, Housing Choice/Housing Choice vouchers, Public Housing, Section 42 Housing))
☐ Healthcare assistance
☐ Disability Assistance (including VA disability assistance)
☐ Work First (TANF)
☐ Other

If you selected Other, Please explain the public assistance you lost.

____

What happened that caused you to lose this assistance?

____

Part B. Work and Finances

What is your average monthly income from wages (i.e. pay for the jobs you work)?

- ◯ Less than $500
- ◯ $501 to $1,000
- ◯ $1,001 to $1,500
- ◯ $1,501 to $2,000
- ◯ $2,001 to $2,500
- ◯ More than $2,500

Do you have a physical health condition or disability that affects the number of hours of work you perform in a week?

- ◯ Yes
- ◯ No

Do you have mental health condition or disability that affects the number of hours of work you perform in a week?

- ◯ Yes
- ◯ No

Which one of the following best describes how well you are with managing money these days:

- ◯ Living comfortably
- ◯ Doing okay
- ◯ Just getting by
- ◯ Finding it difficult to get by

Compared to one year (12 months) ago, would you say that you (and your family living with you) are better off, the same, or worse off with money?

- ◯ Much better off
- ◯ Somewhat better off
- ◯ About the same
- ◯ Somewhat worse off
- ◯ Much worse off

Which of the following hardships did you or your family experience in the last year (12 months)? (Check all that apply)

- ☐ I lost a job
- ☐ I had my hours reduced
- ☐ I changed jobs
- ☐ I was suspended from work without pay
- ☐ I had a medical procedure or health emergency
- ☐ I experienced the death of another income earner
- ☐ I began caring for a loved one
- ☐ I was incarcerated
- ☐ I was arrested/charged with a crime
- ☐ I experienced divorce/separation
- ☐ I experienced the birth of a child
- ☐ I and/or my family lost transportation (vehicle repossessed or totaled, bus service cut etc...)
- ☐ I and/or my family experienced homelessness (homeless or displaced from home)
- ☐ I and/or my family received a foreclosure or eviction
- ☐ I and/or my family experienced the death of a loved one
- ☐ My partner/spouse lost their job
- ☐ My partner/spouse had their hours reduced
- ☐ Other
- ☐ None of the above

If you selected Other, please specify:

____

In the last year (12 months), did you (or your husband/wife/partner) do any of the following? (check all that apply)

- ☐ Apply for a credit card or respond to a pre-approved credit card offer
- ☐ Request an increase in the credit limit of a credit card
- ☐ Apply for a mortgage or home-based loan
- ☐ Request to refinance a mortgage
- ☐ Apply for an auto loan
- ☐ Apply for a student loan
- ☐ Request an increase in the limit of an existing loan (other than a credit card)

How do you pay your bills? (Check all that apply)

- ☐ Electronic payment from bank
- ☐ Personal check
- ☐ Debit card
- ☐ Credit card
- ☐ Bank money order
- ☐ Cash
- ☐ Non-bank money order
- ☐ Pre-paid card
- ☐ Other

What other method do you use to pay your bills?

____

Have you used any of the following services in the last year (12 months)?
(Check all that apply)

- ☐ Pawn Shop
- ☐ Pay Day Loan
- ☐ Auto Title Loan
- ☐ Paycheck Advance Service
- ☐ Tax Refund Anticipation Loan
- ☐ Borrowed money from a friend, family or community member
- ☐ Got a loan from my employer
- ☐ None of the above

If you were faced with a $400 emergency expense today, how would you most likely cover it?

(Check all that apply)

- ☐ Use money from recent paycheck or savings
- ☐ Borrow money from a friend, family or community member
- ☐ Put it on my credit card and pay it off over time
- ☐ Sell something you own
- ☐ Delay payment on other bills or expenses
- ☐ Pawn Shop
- ☐ Pay Day Loan or Paycheck Advance Service
- ☐ Auto Title Loan
- ☐ Tax Refund Anticipation Loan
- ☐ Ask my employer for a loan
- ☐ I do not know how I would cover this expense
- ☐ Other

If you selected Other, Please explain the way you would cover this emergency expense

____

If you were faced with a $500 emergency expense today, how would you most likely cover it?<br><br>(Check all that apply)	☐ Use money from recent paycheck or savings<br>☐ Borrow money from a friend, family or community member<br>☐ Put it on my credit card and pay it off over time<br>☐ Sell something you own<br>☐ Delay payment on other bills or expenses<br>☐ Pawn Shop<br>☐ Pay Day Loan or Paycheck Advance Service<br>☐ Auto Title Loan<br>☐ Tax Refund Anticipation Loan<br>☐ Ask my employer for a loan<br>☐ I do not know how I would cover this expense<br>☐ Other
If you selected Other, Please explain the way you would cover this emergency expense	__________________________________
If you were faced with a $600 emergency expense today, how would you most likely cover it?<br><br>(Check all that apply)	☐ Use money from recent paycheck or savings<br>☐ Borrow money from a friend, family or community member<br>☐ Put it on my credit card and pay it off over time<br>☐ Sell something you own<br>☐ Delay payment on other bills or expenses<br>☐ Pawn Shop<br>☐ Pay Day Loan or Paycheck Advance Service<br>☐ Auto Title Loan<br>☐ Tax Refund Anticipation Loan<br>☐ Ask my employer for a loan<br>☐ I do not know how I would cover this expense<br>☐ Other
If you selected Other, please explain the way you would cover this emergency expense	__________________________________
In the last year (12 months), have you put off paying a bill because you didn't have enough money to pay it when it was due?	○ Yes<br>○ No
Do you have an account with a bank or credit union?	○ Yes<br>○ No

Part C. Accessing Food

What is the name and location of the store where MOST of the food for your household is purchased?

Name
Location (If you do not know the address you can list cross streets and nearby landmarks)
City

____

What is the name and location of the store where MOST of the food for your household is purchased?

Store Name: ___________________________

Store Location (address or nearby intersection, neighborhood, or landmark): ___________________________

Store Location (City): ___________________________

Who usually shops for most of the food for your household?

(Choose more than one if the task is evenly split)

- ☐ Me
- ☐ Spouse/Partner
- ☐ Child/Children
- ☐ Other adult in the home
- ☐ Other

If you selected Other, please specify: ___________________________

How do you usually get to the store where you get most of your food?

- ○ Drive myself in a vehicle
- ○ Family member/friends drive me in their vehicle
- ○ Take public transportation (e.g. bus)
- ○ Walk
- ○ Taxi, Uber, or other car service
- ○ Bicycle
- ○ Other

If you selected Other, please specify: ___________________________

Please indicate how often each statement below was true for your household in the last year (12 months):

	Often true	Sometimes true	Never true
The food that we bought just didn't last and we didn't have money to get more.	◯	◯	◯
We couldn't afford to eat balanced meals.	◯	◯	◯

In the last year (12 months), did you or other adults in the household ever cut the size of your meal or skip meals because there was not enough money for food?
◯ Yes
◯ No
◯ Don't Know

How often did this happen?
◯ Almost every month
◯ Some months but not every month
◯ 1 or 2 months

In the last year (12 months), did you ever eat less than you felt you should because there wasn't enough money for food?
◯ Yes
◯ No
◯ Don't Know

In the last year (12 months), were you ever hungry but didn't eat because there wasn't enough money for food?
◯ Yes
◯ No
◯ Don't Know

In the last year (12-months) have you visited a food shelf (food bank or food pantry)?
◯ Yes
◯ No

Part D. Your Health

In general, would you say your health is:

- ◯ Excellent
- ◯ Very good
- ◯ Good
- ◯ Fair
- ◯ Poor

Are you currently pregnant?

- ◯ Yes
- ◯ No

Do you have health insurance? If so, please indicate what type(s) of health insurance you currently have.

(Check all that apply)

- ☐ Medicaid
- ☐ Medicare
- ☐ Health insurance plan offered through your employer
- ☐ Health insurance through your parent's insurance plan/employer
- ☐ Health insurance plan obtained through Healthcare.gov or MNsure (ACA Exchange)
- ☐ Private Insurance Plan (not obtained through insurance exchange/MNSure)
- ☐ Veterans' Administration (VA) Health Benefits
- ☐ Indian Health Services (IHS) tribal and/or urban Indian health programs
- ☐ Uninsured
- ☐ Other

If you checked Other, please specify:

____

When was the last time you visited a doctor, physician assistant or nurse in a clinic or medical office?

Do not include times you were hospitalized overnight or visits to the hospital emergency room.

- ◯ Within the last 12 months
- ◯ Within the last 1-2 years
- ◯ Within the last 2-5 years
- ◯ More than 5 years ago
- ◯ Never

In the last year (12 months), did you have an illness, injury, or condition that needed care right away in a clinic, emergency room, or doctor's office?

- ◯ Yes
- ◯ No

In the last year (12 months), when you needed care right away, how often did you get care as soon as you thought you needed it?

- ◯ Never
- ◯ Sometimes
- ◯ Usually
- ◯ Always

In the last year (12 months), not counting the times you needed care right away, did you make appointments for your health care at a doctor's office or clinic?

- ◯ Yes
- ◯ No

In the last year (12 months), how often did doctors or other health providers listen carefully to you?

- ◯ Never
- ◯ Sometimes
- ◯ Usually
- ◯ Always

In the last year (12 months), how often did doctors or other health providers show respect for what you had to say?

- ◯ Never
- ◯ Sometimes
- ◯ Usually
- ◯ Always

In the last year (12 months), how often did doctors or other health providers spend enough time with you?

- ◯ Never
- ◯ Sometimes
- ◯ Usually
- ◯ Always

Please check one of the boxes to indicate how strongly you agree or disagree with each statement.

	Strongly Disagree	Somewhat Disagree	Neutral	Somewhat Agree	Strongly Agree
I'm healthy enough that I really don't need health insurance.	○	○	○	○	○
Health insurance is not worth the money it costs.	○	○	○	○	○
I'm more likely to take risks than the average person.	○	○	○	○	○
I can overcome illness without help from a medically trained person.	○	○	○	○	○

Considering a 7-Day period (a week), how many times on average do you do the following kinds of exercise for more than 15 minutes during your free time:

STRENUOUS EXERCISE<br>(Heart beats rapidly)<br>Examples: running, jogging, hockey, football, soccer, basketball, cross-country skiing, roller blading, vigorous swimming, vigorous bicycling	________________<br>(_____ times/week)
MODERATE EXERCISE<br>(Not exhausting)<br>Examples: fast walking, baseball, tennis, easy bicycling, badminton, easy swimming, downhill skiing, recreational dancing	________________<br>(_____ times/week)
MILD EXERCISE<br>(Minimal effort)<br>Examples: easy walking, yoga, fishing, bowling, golf	________________<br>(_____ times/week)
How much time do you usually spend sitting or reclining on a typical day?<br><br>Include time spent sitting at work, at home, getting to and from places (i.e. in a car, bus or train). Do not include time spent sleeping.	________________<br>(Hours)
How much time did you spend last week playing video games?	________________<br>(Hours)
What time do you usually go to bed (turn off the lights to sleep)?	◯ 1 AM ◯ 2 AM ◯ 3 AM<br>◯ 4 AM ◯ 5 AM ◯ 6 AM<br>◯ 7 AM ◯ 8 AM ◯ 9 AM<br>◯ 10 AM ◯ 11 AM ◯ 12 PM (noon)<br>◯ 1 PM ◯ 2 PM ◯ 3 PM<br>◯ 4 PM ◯ 5 PM ◯ 6 PM<br>◯ 7 PM ◯ 8 PM ◯ 9 PM<br>◯ 10 PM ◯ 11 PM ◯ 12 AM (midnight)

What time do you usually get out of bed?	◯ 1 AM ◯ 2 AM ◯ 3 AM ◯ 4 AM ◯ 5 AM ◯ 6 AM ◯ 7 AM ◯ 8 AM ◯ 9 AM ◯ 10 AM ◯ 11 AM ◯ 12 PM (noon) ◯ 1 PM ◯ 2 PM ◯ 3 PM ◯ 4 PM ◯ 5 PM ◯ 6 PM ◯ 7 PM ◯ 8 PM ◯ 9 PM ◯ 10 PM ◯ 11 PM ◯ 12 AM (midnight)
Please select one of the following:	◯ Current smoker ◯ Quit less than 12 months ago ◯ Quit more than 12 months ago ◯ Never smoked

What product(s) do you smoke most regularly? (Check all that apply)

☐ Cigarettes
☐ Cigars, cigarillos, or little cigars
☐ E-cigarettes (Juul, e-cig, vaping pen)
☐ Tobacco in a hookah or waterpipe
☐ Pipes filled with tobacco (not waterpipes)
☐ Bidis (small brown cigarettes wrapped in a leaf)
☐ Marijuana
☐ Other

If you selected Other, Please explain the other product you smoke? ______________________________

Please indicate how often each statement below was true for you in the last month (30 days)

	Never	Almost Never	Sometimes	Fairly Often	Very Often
In the last month (30 days), how often have you felt unable to control the important things in your life?	○	○	○	○	○
In the last month (30 days), how often have you felt confident about your ability to handle your personal problems?	○	○	○	○	○
In the last month (30 days), how often have you felt that things were going your way?	○	○	○	○	○
In the last month (30 days), how often have you felt difficulties were piling up so high that you could not overcome them?	○	○	○	○	○

Part E. Dietary Screener Questionnaire

These questions are about foods you ate or drank during the past month (30 days). When answering, please include meals and snacks at home, at work or school, in restaurants and anyplace else.

During the past month (30 days), how often did you eat hot or cold cereals?	◯ Never ◯ 1 time last month<br>◯ 2-3 times last month<br>◯ 1 time per week ◯ 2 times per week<br>◯ 3-4 times per week ◯ 5-6 times per week ◯ 1 time per day<br>◯ 2 or more times per day
During the past month (30 days), what kind of cereal did you usually eat? (For example, Honey Nut Cheerios, grits, oatmeal, Cinnamon Toast Crunch, Froot Loops, etc. Please be as DETAILED as possible.)	__________________________________
If there was another kind of cereal that you usually ate during the past month (30 days), what kind was it?	__________________________________<br>(Skip if no other kind of cereal)
During the past month (30 days), how often did you drink regular soda or pop that contains sugar? Do not include diet soda.	◯ Never ◯ 1 time last month<br>◯ 2-3 times last month<br>◯ 1 time per week ◯ 2 times per week<br>◯ 3-4 times per week ◯ 5-6 times per week ◯ 1 time per day<br>◯ 2-3 times per day ◯ 4-5 times per day<br>◯ 6 or more times per day
During the past month (30 days), how often did you drink 100% pure fruit juices such as orange, mango, apple, grape and pineapple juices?<br><br>Do not include fruit-flavored drinks with added sugar or fruit juice you made at home and added sugar to.	◯ Never ◯ 1 time last month<br>◯ 2-3 times last month<br>◯ 1 time per week ◯ 2 times per week<br>◯ 3-4 times per week ◯ 5-6 times per week ◯ 1 time per day<br>◯ 2-3 times per day ◯ 4-5 times per day<br>◯ 6 or more times per day
During the past month (30 days), how often did you drink coffee or tea that had sugar or honey added to it?<br><br>Include coffee and tea you sweetened yourself and presweetened tea and coffee drinks such as Arizona Iced Tea and Frappuccino. Do not include artificially sweetened coffee or diet tea.	◯ Never ◯ 1 time last month<br>◯ 2-3 times last month<br>◯ 1 time per week ◯ 2 times per week<br>◯ 3-4 times per week ◯ 5-6 times per week ◯ 1 time per day<br>◯ 2-3 times per day ◯ 4-5 times per day<br>◯ 6 or more times per day
During the past month (30 days), how often did you drink sweetened fruit drinks, sports or energy drinks, such as Kool-Aid, lemonade, Hi-C, cranberry drink, Gatorade, Red Bull or Vitamin Water?<br><br>Include fruit juices you made at home and added sugar to. Do not include diet drinks or artificially sweetened drinks.	◯ Never ◯ 1 time last month<br>◯ 2-3 times last month<br>◯ 1 time per week ◯ 2 times per week<br>◯ 3-4 times per week ◯ 5-6 times per week ◯ 1 time per day<br>◯ 2-3 times per day ◯ 4-5 times per day<br>◯ 6 or more times per day

During the past month (30 days), how often did you eat fruit?<br><br>Include fresh, frozen, or canned fruit. Do not include juices.	◯ Never ◯ 1 time last month<br>◯ 2-3 times last month<br>◯ 1 time per week ◯ 2 times per week<br>◯ 3-4 times per week ◯ 5-6 times per week ◯ 1 time per day<br>◯ 2 or more times per day
During the past month (30 days), how often did you eat a green leafy or lettuce salad, with or without other vegetables?	◯ Never ◯ 1 time last month<br>◯ 2-3 times last month<br>◯ 1 time per week ◯ 2 times per week<br>◯ 3-4 times per week ◯ 5-6 times per week ◯ 1 time per day<br>◯ 2 or more times per day
During the past month (30 days), how often did you eat any kind of fried potatoes, including French fries, home fries, or hash brown potatoes?	◯ Never ◯ 1 time last month<br>◯ 2-3 times last month<br>◯ 1 time per week ◯ 2 times per week<br>◯ 3-4 times per week ◯ 5-6 times per week ◯ 1 time per day<br>◯ 2 or more times per day
During the past month (30 days), how often did you eat any other kind of potatoes, such as baked, boiled, mashed potatoes, sweet potatoes, or potato salad?	◯ Never ◯ 1 time last month<br>◯ 2-3 times last month<br>◯ 1 time per week ◯ 2 times per week<br>◯ 3-4 times per week ◯ 5-6 times per week ◯ 1 time per day<br>◯ 2 or more times per day
During the past month (30 days), how often did you eat refried beans, baked beans, beans in soup, pork and beans or any other type of cooked dried beans?<br><br>Do not include green beans.	◯ Never<br>◯ 1 time last month<br>◯ 2-3 times last month<br>◯ 1 time per week<br>◯ 2 times per week<br>◯ 3-4 times per week<br>◯ 5-6 times per week<br>◯ 1 time per day<br>◯ 2 or more times per day
During the past month (30 days), how often did you eat brown rice or other cooked whole grains, such as bulgur, cracked wheat, or millet? Do not include white rice.	◯ Never ◯ 1 time last month<br>◯ 2-3 times last month<br>◯ 1 time per week ◯ 2 times per week<br>◯ 3-4 times per week ◯ 5-6 times per week ◯ 1 time per day<br>◯ 2 or more times per day
During the past month (30 days), not including the foods you just reported on (i.e., green salads, potatoes, cooked dried beans), how often did you eat other vegetables?	◯ Never ◯ 1 time last month<br>◯ 2-3 times last month<br>◯ 1 time per week ◯ 2 times per week<br>◯ 3-4 times per week ◯ 5-6 times per week ◯ 1 time per day<br>◯ 2 or more times per day

During the past month (30 days), how often did you have Mexican-type salsa made with tomato?	◯ Never ◯ 1 time last month ◯ 2-3 times last month ◯ 1 time per week ◯ 2 times per week ◯ 3-4 times per week ◯ 5-6 times per week ◯ 1 time per day ◯ 2 or more times per day
During the past month (30 days), how often did you eat pizza? Include frozen pizza, fast food pizza, and homemade pizza.	◯ Never ◯ 1 time last month ◯ 2-3 times last month ◯ 1 time per week ◯ 2 times per week ◯ 3-4 times per week ◯ 5-6 times per week ◯ 1 time per day ◯ 2 or more times per day
During the past month (30 days), how often did you have tomato sauces such as with spaghetti or noodles or mixed into foods such as lasagna? Do not include tomato sauce on pizza.	◯ Never ◯ 1 time last month ◯ 2-3 times last month ◯ 1 time per week ◯ 2 times per week ◯ 3-4 times per week ◯ 5-6 times per week ◯ 1 time per day ◯ 2 or more times per day
During the past month (30 days), how often did you eat whole grain bread including toast, rolls and in sandwiches? Whole grain breads include whole wheat, rye, oatmeal and pumpernickel. Do not include white bread.	◯ Never ◯ 1 time last month ◯ 2-3 times last month ◯ 1 time per week ◯ 2 times per week ◯ 3-4 times per week ◯ 5-6 times per week ◯ 1 time per day ◯ 2 or more times per day
During the past month (30 days), how often did you eat chocolate or any other types of candy? Do not include sugar-free candy.	◯ Never ◯ 1 time last month ◯ 2-3 times last month ◯ 1 time per week ◯ 2 times per week ◯ 3-4 times per week ◯ 5-6 times per week ◯ 1 time per day ◯ 2 or more times per day
During the past month (30 days), how often did you eat doughnuts, sweet rolls, Danish, muffins, pan dulce, or pop-tarts? Do not include sugar-free items.	◯ Never ◯ 1 time last month ◯ 2-3 times last month ◯ 1 time per week ◯ 2 times per week ◯ 3-4 times per week ◯ 5-6 times per week ◯ 1 time per day ◯ 2 or more times per day
During the past month (30 days), how often did you eat cookies, cake, pie, or brownies? Do not include sugar-free kinds.	◯ Never ◯ 1 time last month ◯ 2-3 times last month ◯ 1 time per week ◯ 2 times per week ◯ 3-4 times per week ◯ 5-6 times per week ◯ 1 time per day ◯ 2 or more times per day

During the past month (30 days), how often did you eat ice cream or other frozen desserts? Do not include sugar-free kinds.	◯ Never ◯ 1 time last month<br>◯ 2-3 times last month<br>◯ 1 time per week ◯ 2 times per week<br>◯ 3-4 times per week ◯ 5-6 times per week ◯ 1 time per day<br>◯ 2-3 times per day ◯ 4-5 times per day<br>◯ 6 or more times per day
During the past month (30 days), how often did you eat popcorn?	◯ Never ◯ 1 time last month<br>◯ 2-3 times last month<br>◯ 1 time per week ◯ 2 times per week<br>◯ 3-4 times per week ◯ 5-6 times per week ◯ 1 time per day<br>◯ 2-3 times per day ◯ 4-5 times per day<br>◯ 6 or more times per day
General Survey Notes	______________________________

Part G. Self-reported Weight

Note: We will not measure height in Year 3 and will use the Year 2 value as the default.

What is your current weight?

____
(Based on your best estimate)

[height_2]

Part H. Significant Events of 2020 We would like to learn about your experiences this year in light of the COVID-19 pandemic, stay-at-home order, and recent protests. We will ask a number of questions about how these events may have affected your employment, health, and food access.

In Minneapolis, the COVID-19 stay-at-home order started on March 16th and ended on June 1st. In Raleigh, the COVID-19 stay-at-home order started on March 26th and ended on May 22nd. In June, the world responded with outrage, grief, and protest to the murder of George Floyd. Uprisings in Minneapolis and Raleigh interrupted routine operations at many places of employment. Please answer the following questions keeping the significant events of this period from March to June in mind.

EMPLOYMENT

Which of these options best describe your employment situation during the stay-at-home order?

- ◯ Unemployed throughout this period
- ◯ Worked at same job/employer throughout this period
- ◯ Got a new job
- ◯ Lost my job
- ◯ Quit my job (e.g. because I didn't feel safe)

Did you experience any of the following? Select all that apply.

- ☐ Reduction in hours worked
- ☐ Increase in hours worked
- ☐ Reduction in hourly wage or salary
- ☐ Increase in hourly wage or salary (e.g., hazard pay)
- ☐ Furlough (Defined as temporary leave of absence after which your employer wants you back in your position .)
- ☐ Laid off
- ☐ None of the above

If you were employed during the stay-at-home order, was your employment later affected by the protests and related events in June?

(Related events, e.g. businesses closed and some areas of the city experienced damage and disruptions to normal operations)

- ◯ Yes
- ◯ No

In what ways was your employment affected? Select all that apply.

- ☐ I was temporarily unable to go to work during (or around) the protests
- ☐ My place of employment was closed temporarily
- ☐ My place of employment was damaged
- ☐ My place of employment closed and did not reopen
- ☐ I permanently lost a job
- ☐ Other

If you selected other, please specify: ________________________

Did your job duties change during the stay-at-home order?	◯ Yes<br>◯ No
How did your job duties change during the stay-at-home order?	__________________________________
Did your job require you to report for work outside of the home during the stay-at-home order?	◯ I worked outside of the home.<br>◯ I worked from home.
Did your employer take measures to protect you from health risks due to the pandemic?	◯ Yes<br>◯ No<br>((i.e. Did your workplace make changes such as installing plastic barriers for cashiers, provision of face masks, surgical gloves, or other coverings for employees, or implement other measures?))
Did you have access to paid sick leave as part of your compensation and benefits from your employer?	◯ Yes<br>◯ No<br>◯ Do not know
Did you take paid leave because your were sick or had to take care of someone who was sick?	◯ I took paid sick leave.<br>◯ I did not need to take any sick leave<br>◯ I took unpaid leave because of illness as I do not have paid sick leave<br>◯ I had to leave my job or was let go because of illness

HEALTHCARE ACCESS

Did you try to get tested for COVID-19?	◯ Yes<br>◯ No
Did you have access to testing as early as you wanted it?	◯ Yes<br>◯ No
How did you pay for the test?	◯ My insurance covered all the costs.<br>◯ My insurance paid for some of the costs and I paid a co-payment.<br>◯ I was responsible for the full amount of the test out-of-pocket.<br>◯ My employer paid for the test.<br>◯ The test was free.<br>◯ Don't know.<br>◯ Did not get tested
If you needed medical treatment for COVID-19 (e.g. see a doctor, get a chest x-ray, stay in the hospital, etc.), did you have access to the treatment you needed?	◯ Yes<br>◯ No<br>◯ Did not need treatment
How did you pay for the cost of your COVID-19 treatment?	◯ My insurance covered all the costs.<br>◯ My insurance paid for some of the costs and I paid a co-payment.<br>◯ I was responsible for the full amount of the treatment out-of-pocket.<br>◯ My employer paid for the treatment.<br>◯ Don't know.

FINANCES DURING COVID-19

How did your household income and expenses differ during the stay at home period?

- ◯ No significant difference
- ◯ Had less income
- ◯ Had more income

(Think about all sources of money or other resources coming into the household during the stay at home period, including wages and unemployment benefits.)

How did your household expenses differ during the stay at home period? Select all that apply.

- ☐ Expenses were lower
- ☐ Expenses stayed the same
- ☐ Expenses went up
- ☐ I accrued more debt than normal

Did you receive financial support from any of these sources during the stay-at-home order?

	Yes	No	Don't know
Unemployment insurance	○	○	○
Money from church or faith-based organization / community	○	○	○
Money from family or friends	○	○	○
Money from crowdsourcing platforms (e.g. GoFundMe)	○	○	○
Government stimulus check ($1,200 per adult and $500 per child)	○	○	○

How did you use the money from the stimulus check? Select all that apply.

- ☐ Used money to pay rent/mortgage/hotel expenses
- ☐ Used to pay other bills
- ☐ Used money to buy food needed for me or my household
- ☐ Used money to pay for needed medical care
- ☐ Used money to treat my family to something
- ☐ Don't remember
- ☐ Other

If you responded Other, please specify. ______________________________

ACCESSING FOOD

During the stay-at-home order were there times when you:	☐ Couldn't afford to buy enough food<br>☐ Couldn't afford to eat balanced meals<br>☐ Had to cut the size of meals or skip meals because there wasn't enough money for food<br>☐ Were hungry because there wasn't enough food to eat<br>☐ None of the above
Which of the following food support resources did you or anyone in your household use for the first time during the stay-at-home order? Select all that apply.	☐ SNAP/EBT (formerly food stamps)<br>☐ WIC<br>☐ School meal sites (e.g. School Lunch or Breakfast)<br>☐ Food shelf or pantry<br>☐ Soup kitchen (e.g. distribution of prepared or frozen meals)<br>☐ Garden (e.g. backyard, community)<br>☐ Other<br>☐ None of the above
If you responded Other, please specify.	__________________________________

During the stay-at-home order, were your typical food shopping activities affected in any of the following ways?

	Yes	No
Unable to buy needed foods at grocery store (or other place you buy food) because food was out of stock	○	○
Affordable size or brand of food was not available	○	○
Food prices higher than usual	○	○
Closure of a grocery store or other place that you typically buy food at	○	○
Closure of a food shelf/pantry you typically rely on	○	○
Limited availability of foods at a food shelf/pantry you typically rely on	○	○
Started shopping for groceries online	○	○
Bought more take-out or prepared food than usual	○	○
Needed to buy more food than normal due to school closures	○	○

Due to the protests in June were your food shopping activities affected in any of the following ways?

	Yes	No
Small food store where you typically buy food was damaged and remains closed.	○	○
Large grocery store or big box store where you typically buy food was damaged and remains closed.	○	○
I relied on free food that was given out near the areas of the protests	○	○
Other	○	○

If you selected other, please provide details. __________________________________

During the stay-at-home order, how was your diet different? Select all that apply.

- ☐ Ate less healthy
- ☐ Ate more healthy
- ☐ Did not change
- ☐ Other (please specify):

If you responded Other, please specify details: __________________________________

HEALTH AND STRESS

Where did you live during the stay-at-home order?	☐ My home<br>☐ A friend or family member's home<br>☐ A hotel<br>☐ A shelter<br>☐ Other
If you selected Other, please specify:	__________________________________
How much stress did the stay-at-home order (including guidelines for social distancing) cause you?	○ A lot<br>○ Some<br>○ A little<br>○ Not at all
Which of the following did you experience during the stay-at-home order? Select all that apply.	☐ I felt disconnected or isolated from others<br>☐ My relationships with people around me became weaker or more difficult<br>☐ I felt a greater sense of community/connection with others<br>☐ The things I do felt meaningless<br>☐ Experienced personal growth/became a better version of myself<br>☐ My relationships with others became stronger<br>☐ I felt a greater sense of meaning in life<br>☐ I felt hopeless about the future of the world<br>☐ I felt stress or worry when caring for elders<br>☐ None of the above
The following are behaviors that people reported engaging in during the stay-at-home order. Please select all the following behaviors you engaged in during that time.	☐ I argued excessively with others in my home<br>☐ I drank alcohol more often than usual<br>☐ I ate more junk food like snack chips, candy, and cookies<br>☐ I set health-related goals<br>☐ I developed new hobbies or interests<br>☐ I slept at irregular hours or slept less or more than desired<br>☐ I went days without leaving the house (even for fresh air)<br>☐ I reconnected with people outside my home by text, phone or video chat<br>☐ I gained undesired weight<br>☐ None of the above

2021 (T4) Wages Participant Survey

Please take this survey.
You may open the survey in your web browser by clicking the link below:
[survey-link]
If the link above does not work, try copying the link below into your web browser:
[survey-url]
This link is unique to you and should not be forwarded to others.

How many adults (age 18 or older) currently live in your household (including yourself)?

- ○ 1
- ○ 2
- ○ 3
- ○ 4
- ○ 5 or more

How many children (under the age of 18) currently live in your household, including children who live there part-time?

- ○ 0
- ○ 1
- ○ 2
- ○ 3
- ○ 4
- ○ 5 or more

Please list the following information for each child living in the household:

Child 1
Age

____
(Years)

Child 1 Age

(Enter months if under age 1)

Years

____

Months

____

Child 1 Weight

____
(Pounds (lbs))

Child 1 Height

____
(Feet / Inches)

Child 2 Age

(Enter months if under age 1)

Years

____

Months

____

Child 2
Age

____
(Years)

Child 2 Weight

____
(Pounds (lbs))

Child 2 Height

____
(Feet / Inches)

Child 3
Age

____
(Years)

Child 3 Age

(enter months if under age 1)

Years

____

Months ________________

Child 3 Weight ________________ (Pounds (lbs))

Child 3 Height ________________ (Feet / Inches)

Child 4
Age ________________ (Years)

Child 4 Age

(Enter months if under age 1)

Years ________________

Months ________________

Child 4 Weight ________________ (Pounds (lbs))

Child 4 Height ________________ (Feet / Inches)

Child 5
Age ________________ (Years)

Child 5 Age

(enter months if under age 1)

Years ________________

Months ________________

Child 5 Weight ________________ (Pounds (lbs))

Child 5 Height ________________ (Feet / Inches)

How did you determine the child or children's height or weight?
◯ Pediatrician or health care provider records
◯ School records
◯ Measured at home
◯ My best guess
◯ Other

If you selected Other, please specify:
____

Are you registered to vote?
◯ Yes
◯ No

Did you vote in the last local election, like for mayor or city council member?
◯ Yes
◯ No

Did you vote in the last general election, like for state representatives, senators and president?
◯ Yes
◯ No

During the past year did you attend a political protest, march or demonstration?
◯ Yes
◯ No

During the past year did you contact a public official?
◯ Yes
◯ No

During the past year did you work for a candidate or campaign?
◯ Yes
◯ No

In the last year (12 months), was there a time when you were not able to pay the mortgage or rent on time?
◯ Yes
◯ No

In the last year (12 months), how many places have you lived?
◯ 0
◯ 1
◯ 2
◯ 3
◯ More than 3

In the last year (12 months), was there a time when you did not have a steady place to sleep or slept in a shelter (including now)?
◯ Yes
◯ No

What is the highest grade or level of school you have completed?

◯ Less than High School
◯ Some High School
◯ High School Diploma
◯ Associate/Technical Degree
◯ Some College
◯ Bachelor's Degree
◯ Graduate Degree

When did you complete this schooling?
____
(Year (YYYY))

Have you completed any of the following types of trainings/career development activities in the last year (12 months)?
(Check all that apply)

☐ A training, workshop, seminar or professional event required by your employer
☐ A training, workshop, seminar or professional event not required by your employer
☐ Licensure or certification for your occupation
☐ A training, workshop, seminar or professional development event to help you change to a new occupation
☐ Other
☐ None of the above

If you selected Other, please specify:

____

What was your annual household income before taxes (from all sources), last year?

◯ Less than $5,000
◯ $5,001 to $10,000
◯ $10,001 to $20,000
◯ $20,001 to $30,000
◯ $30,001 to $40,000
◯ $40,001 to $50,000
◯ More than $50,000

Do you own or have access to a vehicle for reliable transportation?

◯ Yes
◯ No

In the last month (30 days) did you or anyone in your household receive any of the following? (Check Yes or No for EACH program)

	Yes	No	Not Sure
WIC (Women, Infants, and Children program)	◯	◯	◯
Food stamps (SNAP or EBT)	◯	◯	◯
Free or reduced price school lunch	◯	◯	◯
MFIP (Minnesota Family Investment Program) (You may receive one or more of the following together or separately: Cash, Food support, childcare assistance, housing grant.)	◯	◯	◯
State housing subsidy (e.g., Bridges Housing Subsidy, HUD Rental Assistance, Housing Choice/Housing Choice vouchers, Public Housing, Section 42 Housing)	◯	◯	◯
Disability assistance (including VA disability assistance)	◯	◯	◯
Work First (Temporary Assistance for the Needy Families, TANF)	◯	◯	◯

How much did your household receive in SNAP benefits in the last month (30 days)?	◯ I do not receive any food stamps or SNAP benefits<br>◯ $1 - $25<br>◯ $26 - $50<br>◯ $51 - $75<br>◯ $76 - $100<br>◯ $101 - $150<br>◯ $151 - $250<br>◯ $251 - $500<br>◯ $501 - $750<br>◯ More than $750
Did your wages (dollars per hour) increase in the last year (12 months)?	◯ Yes<br>◯ No
If Yes, has your increase in wages made you worry about losing any public assistance? (housing subsidy, SNAP, WIC, MFIP, healthcare assistance)	◯ Yes<br>◯ No
Has your worry about losing assistance caused you to make job related changes? (quit job, asked to have hours reduced, got a different job)	◯ Yes<br>◯ No
Please explain the job changes you made because you were worried about losing public assistance.	__________________________________

Have you lost any public assistance in the last year (12 months)?

◯ Yes
◯ No

What public assistance did you lose in the last year (12 months)?

☐ WIC (Women, Infants, and Children program)
☐ Food stamps (SNAP)
☐ Free or reduced price school lunch
☐ MFIP (Minnesota Family Investment Program): Cash, Food support, childcare assistance, housing grant.)
☐ State housing subsidy (e.g., Bridges Housing Subsidy, HUD Rental Assistance, Housing Choice/Housing Choice vouchers, Public Housing, Section 42 Housing))
☐ Healthcare assistance
☐ Disability Assistance (including VA disability assistance)
☐ Work First (TANF)
☐ Other

If you selected Other, Please explain the public assistance you lost.

____

What happened that caused you to lose this assistance?

____

Please indicate your level of agreement with the following statements about using SNAP (or Food Stamp) food benefits since the COVID-19 pandemic?

Since the COVID-19 outbreak:

	Strongly disagree	Disagree	Neutral	Agree	Strongly agree	Don't know
Overall, it is not hard to use SNAP benefits to buy food for our household	◯	◯	◯	◯	◯	◯
SNAP benefits are not enough to meet our household's needs	◯	◯	◯	◯	◯	◯
We cannot use SNAP benefits to pay for groceries ordered online	◯	◯	◯	◯	◯	◯
We are not able to use our full months' worth of SNAP benefits (because, for example, it is hard to go shopping or stores do not have food we need)	◯	◯	◯	◯	◯	◯

Do you have any other comments about using SNAP during the COVID-19 pandemic? (please specify) __________________________________

What is your average monthly income from wages (i.e. pay for the jobs you work)?
- ◯ Less than $500
- ◯ $501 to $1,000
- ◯ $1,001 to $1,500
- ◯ $1,501 to $2,000
- ◯ $2,001 to $2,500
- ◯ More than $2,500

Do you have a physical health condition or disability that affects the number of hours of work you perform in a week?
- ◯ Yes
- ◯ No

Do you have mental health condition or disability that affects the number of hours of work you perform in a week?
- ◯ Yes
- ◯ No

Which one of the following best describes how well you are with managing money these days:
- ◯ Living comfortably
- ◯ Doing okay
- ◯ Just getting by
- ◯ Finding it difficult to get by

Compared to one year (12 months) ago, would you say that you (and your family living with you) are better off, the same, or worse off with money?
- ◯ Much better off
- ◯ Somewhat better off
- ◯ About the same
- ◯ Somewhat worse off
- ◯ Much worse off

Which of the following hardships did you or your family experience in the last year (12 months)? (Check all that apply)

- ☐ I lost a job
- ☐ I had my hours reduced
- ☐ I changed jobs
- ☐ I was suspended from work without pay
- ☐ I had a medical procedure or health emergency
- ☐ I experienced the death of another income earner
- ☐ I began caring for a loved one
- ☐ I was incarcerated
- ☐ I was arrested/charged with a crime
- ☐ I experienced divorce/separation
- ☐ I experienced the birth of a child
- ☐ I and/or my family lost transportation (vehicle repossessed or totaled, bus service cut etc...)
- ☐ I and/or my family experienced homelessness (homeless or displaced from home)
- ☐ I and/or my family received a foreclosure or eviction
- ☐ I and/or my family experienced the death of a loved one
- ☐ My partner/spouse lost their job
- ☐ My partner/spouse had their hours reduced
- ☐ Other
- ☐ None of the above

If you selected Other, please specify:

____

You responded above "I had a medical procedure or health emergency" - Was this related to COVID-19?	◯ Yes<br>◯ No
You responded above "I experienced the death of another income earner - Was this related to COVID-19?	◯ Yes<br>◯ No
You responded above "I began caring for a loved one" - Was this related to COVID-19?	◯ Yes<br>◯ No
You responded above "I experienced the death of a loved one" - Was this related to COVID-19?	◯ Yes<br>◯ No
In the last year (12 months), did you (or your husband/wife/partner) do any of the following? (check all that apply)	☐ Apply for a credit card or respond to a pre-approved credit card offer<br>☐ Request an increase in the credit limit of a credit card<br>☐ Apply for a mortgage or home-based loan<br>☐ Request to refinance a mortgage<br>☐ Apply for an auto loan<br>☐ Apply for a student loan<br>☐ Request an increase in the limit of an existing loan (other than a credit card)
How do you pay your bills? (Check all that apply)	☐ Electronic payment from bank<br>☐ Personal check<br>☐ Debit card<br>☐ Credit card<br>☐ Bank money order<br>☐ Cash<br>☐ Non-bank money order<br>☐ Pre-paid card<br>☐ Other

What other method do you use to pay your bills?

____

Have you used any of the following services in the last year (12 months)?
(Check all that apply)

- ☐ Pawn Shop
- ☐ Pay Day Loan
- ☐ Auto Title Loan
- ☐ Paycheck Advance Service
- ☐ Tax Refund Anticipation Loan
- ☐ Borrowed money from a friend, family or community member
- ☐ Got a loan from my employer
- ☐ None of the above

If you were faced with a $400 emergency expense today, how would you most likely cover it?

(Check all that apply)

- ☐ Use money from recent paycheck or savings
- ☐ Borrow money from a friend, family or community member
- ☐ Put it on my credit card and pay it off over time
- ☐ Sell something you own
- ☐ Delay payment on other bills or expenses
- ☐ Pawn Shop
- ☐ Pay Day Loan or Paycheck Advance Service
- ☐ Auto Title Loan
- ☐ Tax Refund Anticipation Loan
- ☐ Ask my employer for a loan
- ☐ I do not know how I would cover this expense
- ☐ Other

If you selected Other, Please explain the way you would cover this emergency expense

____

If you were faced with a $500 emergency expense today, how would you most likely cover it?

(Check all that apply)

- ☐ Use money from recent paycheck or savings
- ☐ Borrow money from a friend, family or community member
- ☐ Put it on my credit card and pay it off over time
- ☐ Sell something you own
- ☐ Delay payment on other bills or expenses
- ☐ Pawn Shop
- ☐ Pay Day Loan or Paycheck Advance Service
- ☐ Auto Title Loan
- ☐ Tax Refund Anticipation Loan
- ☐ Ask my employer for a loan
- ☐ I do not know how I would cover this expense
- ☐ Other

If you selected Other, Please explain the way you would cover this emergency expense

____

If you were faced with a $600 emergency expense today, how would you most likely cover it?<br><br>(Check all that apply)	☐ Use money from recent paycheck or savings<br>☐ Borrow money from a friend, family or community member<br>☐ Put it on my credit card and pay it off over time<br>☐ Sell something you own<br>☐ Delay payment on other bills or expenses<br>☐ Pawn Shop<br>☐ Pay Day Loan or Paycheck Advance Service<br>☐ Auto Title Loan<br>☐ Tax Refund Anticipation Loan<br>☐ Ask my employer for a loan<br>☐ I do not know how I would cover this expense<br>☐ Other
If you selected Other, please explain the way you would cover this emergency expense	__________________________________
In the last year (12 months), have you put off paying a bill because you didn't have enough money to pay it when it was due?	○ Yes<br>○ No
Do you have an account with a bank or credit union?	○ Yes<br>○ No

Part C. Accessing Food

What is the name and location of the store where MOST of the food for your household is purchased?

Name
Location (If you do not know the address you can list cross streets and nearby landmarks)
City

____

What is the name and location of the store where MOST of the food for your household is purchased?

Store Name: __________________________

Store Location (address or nearby intersection, neighborhood, or landmark): __________________________

Store Location (City): __________________________

Who usually shops for most of the food for your household?

(Choose more than one if the task is evenly split)

- ☐ Me
- ☐ Spouse/Partner
- ☐ Child/Children
- ☐ Other adult in the home
- ☐ Other

If you selected Other, please specify: __________________________

How do you usually get to the store where you get most of your food?

- ○ Drive myself in a vehicle
- ○ Family member/friends drive me in their vehicle
- ○ Take public transportation (e.g. bus)
- ○ Walk
- ○ Taxi, Uber, or other car service
- ○ Bicycle
- ○ Other

If you selected Other, please specify: __________________________

Please indicate how often each statement below was true for your household in the last year (12 months):

	Often true	Sometimes true	Never true
The food that we bought just didn't last and we didn't have money to get more.	◯	◯	◯
We couldn't afford to eat balanced meals.	◯	◯	◯

In the last year (12 months), did you or other adults in the household ever cut the size of your meal or skip meals because there was not enough money for food?
◯ Yes
◯ No
◯ Don't Know

How often did this happen?
◯ Almost every month
◯ Some months but not every month
◯ 1 or 2 months

In the last year (12 months), did you ever eat less than you felt you should because there wasn't enough money for food?
◯ Yes
◯ No
◯ Don't Know

In the last year (12 months), were you ever hungry but didn't eat because there wasn't enough money for food?
◯ Yes
◯ No
◯ Don't Know

In the last year (12-months) have you visited a food shelf (food bank or food pantry)?
◯ Yes
◯ No

Part D. Your Health

In general, would you say your health is:	◯ Excellent<br>◯ Very good<br>◯ Good<br>◯ Fair<br>◯ Poor
Are you currently pregnant?	◯ Yes<br>◯ No
Do you have health insurance? If so, please indicate what type(s) of health insurance you currently have.<br><br>(Check all that apply)	☐ Medicaid<br>☐ Medicare<br>☐ Health insurance plan offered through your employer<br>☐ Health insurance through your parent's insurance plan/employer<br>☐ Health insurance plan obtained through Healthcare.gov or MNsure (ACA Exchange)<br>☐ Private Insurance Plan (not obtained through insurance exchange/MNSure)<br>☐ Veterans' Administration (VA) Health Benefits<br>☐ Indian Health Services (IHS) tribal and/or urban Indian health programs<br>☐ Uninsured<br>☐ Other
If you checked Other, please specify:	__________________________________
When was the last time you visited a doctor, physician assistant or nurse in a clinic or medical office?<br><br>Do not include times you were hospitalized overnight or visits to the hospital emergency room.	◯ Within the last 12 months<br>◯ Within the last 1-2 years<br>◯ Within the last 2-5 years<br>◯ More than 5 years ago<br>◯ Never
In the last year (12 months), did you have an illness, injury, or condition that needed care right away in a clinic, emergency room, or doctor's office?	◯ Yes<br>◯ No
In the last year (12 months), when you needed care right away, how often did you get care as soon as you thought you needed it?	◯ Never<br>◯ Sometimes<br>◯ Usually<br>◯ Always
In the last year (12 months), not counting the times you needed care right away, did you make appointments for your health care at a doctor's office or clinic?	◯ Yes<br>◯ No
In the last year (12 months), how often did doctors or other health providers listen carefully to you?	◯ Never<br>◯ Sometimes<br>◯ Usually<br>◯ Always
In the last year (12 months), how often did doctors or other health providers show respect for what you had to say?	◯ Never<br>◯ Sometimes<br>◯ Usually<br>◯ Always

In the last year (12 months), how often did doctors or other health providers spend enough time with you?

- ◯ Never
- ◯ Sometimes
- ◯ Usually
- ◯ Always

Please check one of the boxes to indicate how strongly you agree or disagree with each statement.

	Strongly Disagree	Somewhat Disagree	Neutral	Somewhat Agree	Strongly Agree
I'm healthy enough that I really don't need health insurance.	○	○	○	○	○
Health insurance is not worth the money it costs.	○	○	○	○	○
I'm more likely to take risks than the average person.	○	○	○	○	○
I can overcome illness without help from a medically trained person.	○	○	○	○	○

Considering a 7-Day period (a week), how many times on average do you do the following kinds of exercise for more than 15 minutes during your free time:

STRENUOUS EXERCISE<br>(Heart beats rapidly)<br>Examples: running, jogging, hockey, football, soccer, basketball, cross-country skiing, roller blading, vigorous swimming, vigorous bicycling	________________<br>(_____ times/week)
MODERATE EXERCISE<br>(Not exhausting)<br>Examples: fast walking, baseball, tennis, easy bicycling, badminton, easy swimming, downhill skiing, recreational dancing	________________<br>(_____ times/week)
MILD EXERCISE<br>(Minimal effort)<br>Examples: easy walking, yoga, fishing, bowling, golf	________________<br>(_____ times/week)
How much time do you usually spend sitting or reclining on a typical day?<br><br>Include time spent sitting at work, at home, getting to and from places (i.e. in a car, bus or train). Do not include time spent sleeping.	________________<br>(Hours)
How much time did you spend last week playing video games?	________________<br>(Hours)
What time do you usually go to bed (turn off the lights to sleep)?	◯ 1 AM ◯ 2 AM ◯ 3 AM<br>◯ 4 AM ◯ 5 AM ◯ 6 AM<br>◯ 7 AM ◯ 8 AM ◯ 9 AM<br>◯ 10 AM ◯ 11 AM ◯ 12 PM (noon)<br>◯ 1 PM ◯ 2 PM ◯ 3 PM<br>◯ 4 PM ◯ 5 PM ◯ 6 PM<br>◯ 7 PM ◯ 8 PM ◯ 9 PM<br>◯ 10 PM ◯ 11 PM ◯ 12 AM (midnight)

What time do you usually get out of bed?

◯ 1 AM ◯ 2 AM ◯ 3 AM
◯ 4 AM ◯ 5 AM ◯ 6 AM
◯ 7 AM ◯ 8 AM ◯ 9 AM
◯ 10 AM ◯ 11 AM ◯ 12 PM (noon)
◯ 1 PM ◯ 2 PM ◯ 3 PM
◯ 4 PM ◯ 5 PM ◯ 6 PM
◯ 7 PM ◯ 8 PM ◯ 9 PM
◯ 10 PM ◯ 11 PM ◯ 12 AM (midnight)

Please select one of the following:

◯ Current smoker ◯ Quit less than 12 months ago ◯ Quit more than 12 months ago ◯ Never smoked

---

What product(s) do you smoke most regularly?
(Check all that apply)

☐ Cigarettes
☐ Cigars, cigarillos, or little cigars
☐ E-cigarettes (Juul, e-cig, vaping pen)
☐ Tobacco in a hookah or waterpipe
☐ Pipes filled with tobacco (not waterpipes)
☐ Bidis (small brown cigarettes wrapped in a leaf)
☐ Marijuana
☐ Other

If you selected Other, Please explain the other product you smoke?

____

Please indicate how often each statement below was true for you in the last month (30 days)

	Never	Almost Never	Sometimes	Fairly Often	Very Often
In the last month (30 days), how often have you felt unable to control the important things in your life?	○	○	○	○	○
In the last month (30 days), how often have you felt confident about your ability to handle your personal problems?	○	○	○	○	○
In the last month (30 days), how often have you felt that things were going your way?	○	○	○	○	○
In the last month (30 days), how often have you felt difficulties were piling up so high that you could not overcome them?	○	○	○	○	○

Part E. Dietary Screener Questionnaire

These questions are about foods you ate or drank during the past month (30 days). When answering, please include meals and snacks at home, at work or school, in restaurants and anyplace else.

During the past month (30 days), how often did you eat hot or cold cereals?	◯ Never ◯ 1 time last month ◯ 2-3 times last month ◯ 1 time per week ◯ 2 times per week ◯ 3-4 times per week ◯ 5-6 times per week ◯ 1 time per day ◯ 2 or more times per day
During the past month (30 days), what kind of cereal did you usually eat? (For example, Honey Nut Cheerios, grits, oatmeal, Cinnamon Toast Crunch, Froot Loops, etc. Please be as DETAILED as possible.)	________________________
If there was another kind of cereal that you usually ate during the past month (30 days), what kind was it?	________________________ (Skip if no other kind of cereal)
During the past month (30 days), how often did you drink regular soda or pop that contains sugar? Do not include diet soda.	◯ Never ◯ 1 time last month ◯ 2-3 times last month ◯ 1 time per week ◯ 2 times per week ◯ 3-4 times per week ◯ 5-6 times per week ◯ 1 time per day ◯ 2-3 times per day ◯ 4-5 times per day ◯ 6 or more times per day
During the past month (30 days), how often did you drink 100% pure fruit juices such as orange, mango, apple, grape and pineapple juices?<br><br>Do not include fruit-flavored drinks with added sugar or fruit juice you made at home and added sugar to.	◯ Never ◯ 1 time last month ◯ 2-3 times last month ◯ 1 time per week ◯ 2 times per week ◯ 3-4 times per week ◯ 5-6 times per week ◯ 1 time per day ◯ 2-3 times per day ◯ 4-5 times per day ◯ 6 or more times per day
During the past month (30 days), how often did you drink coffee or tea that had sugar or honey added to it?<br><br>Include coffee and tea you sweetened yourself and presweetened tea and coffee drinks such as Arizona Iced Tea and Frappuccino. Do not include artificially sweetened coffee or diet tea.	◯ Never ◯ 1 time last month ◯ 2-3 times last month ◯ 1 time per week ◯ 2 times per week ◯ 3-4 times per week ◯ 5-6 times per week ◯ 1 time per day ◯ 2-3 times per day ◯ 4-5 times per day ◯ 6 or more times per day
During the past month (30 days), how often did you drink sweetened fruit drinks, sports or energy drinks, such as Kool-Aid, lemonade, Hi-C, cranberry drink, Gatorade, Red Bull or Vitamin Water?<br><br>Include fruit juices you made at home and added sugar to. Do not include diet drinks or artificially sweetened drinks.	◯ Never ◯ 1 time last month ◯ 2-3 times last month ◯ 1 time per week ◯ 2 times per week ◯ 3-4 times per week ◯ 5-6 times per week ◯ 1 time per day ◯ 2-3 times per day ◯ 4-5 times per day ◯ 6 or more times per day

During the past month (30 days), how often did you eat fruit?<br><br>Include fresh, frozen, or canned fruit. Do not include juices.	◯ Never ◯ 1 time last month<br>◯ 2-3 times last month<br>◯ 1 time per week ◯ 2 times per week<br>◯ 3-4 times per week ◯ 5-6 times per week ◯ 1 time per day<br>◯ 2 or more times per day
During the past month (30 days), how often did you eat a green leafy or lettuce salad, with or without other vegetables?	◯ Never ◯ 1 time last month<br>◯ 2-3 times last month<br>◯ 1 time per week ◯ 2 times per week<br>◯ 3-4 times per week ◯ 5-6 times per week ◯ 1 time per day<br>◯ 2 or more times per day
During the past month (30 days), how often did you eat any kind of fried potatoes, including French fries, home fries, or hash brown potatoes?	◯ Never ◯ 1 time last month<br>◯ 2-3 times last month<br>◯ 1 time per week ◯ 2 times per week<br>◯ 3-4 times per week ◯ 5-6 times per week ◯ 1 time per day<br>◯ 2 or more times per day
During the past month (30 days), how often did you eat any other kind of potatoes, such as baked, boiled, mashed potatoes, sweet potatoes, or potato salad?	◯ Never ◯ 1 time last month<br>◯ 2-3 times last month<br>◯ 1 time per week ◯ 2 times per week<br>◯ 3-4 times per week ◯ 5-6 times per week ◯ 1 time per day<br>◯ 2 or more times per day
During the past month (30 days), how often did you eat refried beans, baked beans, beans in soup, pork and beans or any other type of cooked dried beans?<br><br>Do not include green beans.	◯ Never<br>◯ 1 time last month<br>◯ 2-3 times last month<br>◯ 1 time per week<br>◯ 2 times per week<br>◯ 3-4 times per week<br>◯ 5-6 times per week<br>◯ 1 time per day<br>◯ 2 or more times per day
During the past month (30 days), how often did you eat brown rice or other cooked whole grains, such as bulgur, cracked wheat, or millet? Do not include white rice.	◯ Never ◯ 1 time last month<br>◯ 2-3 times last month<br>◯ 1 time per week ◯ 2 times per week<br>◯ 3-4 times per week ◯ 5-6 times per week ◯ 1 time per day<br>◯ 2 or more times per day
During the past month (30 days), not including the foods you just reported on (i.e., green salads, potatoes, cooked dried beans), how often did you eat other vegetables?	◯ Never ◯ 1 time last month<br>◯ 2-3 times last month<br>◯ 1 time per week ◯ 2 times per week<br>◯ 3-4 times per week ◯ 5-6 times per week ◯ 1 time per day<br>◯ 2 or more times per day

During the past month (30 days), how often did you have Mexican-type salsa made with tomato?	◯ Never ◯ 1 time last month ◯ 2-3 times last month ◯ 1 time per week ◯ 2 times per week ◯ 3-4 times per week ◯ 5-6 times per week ◯ 1 time per day ◯ 2 or more times per day
During the past month (30 days), how often did you eat pizza? Include frozen pizza, fast food pizza, and homemade pizza.	◯ Never ◯ 1 time last month ◯ 2-3 times last month ◯ 1 time per week ◯ 2 times per week ◯ 3-4 times per week ◯ 5-6 times per week ◯ 1 time per day ◯ 2 or more times per day
During the past month (30 days), how often did you have tomato sauces such as with spaghetti or noodles or mixed into foods such as lasagna? Do not include tomato sauce on pizza.	◯ Never ◯ 1 time last month ◯ 2-3 times last month ◯ 1 time per week ◯ 2 times per week ◯ 3-4 times per week ◯ 5-6 times per week ◯ 1 time per day ◯ 2 or more times per day
During the past month (30 days), how often did you eat whole grain bread including toast, rolls and in sandwiches? Whole grain breads include whole wheat, rye, oatmeal and pumpernickel. Do not include white bread.	◯ Never ◯ 1 time last month ◯ 2-3 times last month ◯ 1 time per week ◯ 2 times per week ◯ 3-4 times per week ◯ 5-6 times per week ◯ 1 time per day ◯ 2 or more times per day
During the past month (30 days), how often did you eat chocolate or any other types of candy? Do not include sugar-free candy.	◯ Never ◯ 1 time last month ◯ 2-3 times last month ◯ 1 time per week ◯ 2 times per week ◯ 3-4 times per week ◯ 5-6 times per week ◯ 1 time per day ◯ 2 or more times per day
During the past month (30 days), how often did you eat doughnuts, sweet rolls, Danish, muffins, pan dulce, or pop-tarts? Do not include sugar-free items.	◯ Never ◯ 1 time last month ◯ 2-3 times last month ◯ 1 time per week ◯ 2 times per week ◯ 3-4 times per week ◯ 5-6 times per week ◯ 1 time per day ◯ 2 or more times per day
During the past month (30 days), how often did you eat cookies, cake, pie, or brownies? Do not include sugar-free kinds.	◯ Never ◯ 1 time last month ◯ 2-3 times last month ◯ 1 time per week ◯ 2 times per week ◯ 3-4 times per week ◯ 5-6 times per week ◯ 1 time per day ◯ 2 or more times per day

During the past month (30 days), how often did you eat ice cream or other frozen desserts? Do not include sugar-free kinds.	◯ Never ◯ 1 time last month ◯ 2-3 times last month ◯ 1 time per week ◯ 2 times per week ◯ 3-4 times per week ◯ 5-6 times per week ◯ 1 time per day ◯ 2-3 times per day ◯ 4-5 times per day ◯ 6 or more times per day
During the past month (30 days), how often did you eat popcorn?	◯ Never ◯ 1 time last month ◯ 2-3 times last month ◯ 1 time per week ◯ 2 times per week ◯ 3-4 times per week ◯ 5-6 times per week ◯ 1 time per day ◯ 2-3 times per day ◯ 4-5 times per day ◯ 6 or more times per day
General Survey Notes	____________________________

Part G. Self-reported Weight

Note: If we are not able to measure height in Year 3 and Year 4 we will use the Year 2 value as the default.

What is your current weight?

____
(Based on your best estimate)

[height_2]

Part H. Pandemic Impact

We will ask a number of questions about how the pandemic may have affected your employment, health, and food access.

EMPLOYMENT

Which of these options best describe your employment situation during the last 12 months?	◯ Unemployed throughout this period<br>◯ Worked at same job/employer throughout this period<br>◯ Got a new job<br>◯ Lost my job<br>◯ Quit my job (e.g. because I didn't feel safe)
Did you experience any of the following during the last 12 months? Select all that apply.	☐ Reduction in hours worked<br>☐ Increase in hours worked<br>☐ Reduction in hourly wage or salary<br>☐ Increase in hourly wage or salary (e.g., hazard pay)<br>☐ Furlough (Defined as temporary leave of absence after which your employer wants you back in your position .)<br>☐ Laid off<br>☐ None of the above
In the last 12 months, did your employer take measures to protect you from health risks due to the pandemic?	◯ Yes<br>◯ No<br>((i.e. Did your workplace make changes such as installing plastic barriers for cashiers, provision of face masks, surgical gloves, or other coverings for employees, or implement other measures?))
In the last 12 months, did you have access to paid sick leave as part of your compensation and benefits from your employer?	◯ Yes<br>◯ No<br>◯ Do not know<br>◯ Partially / Some of the time

In the last 12 months, did you take paid leave because you were sick or had to take care of someone who was sick?	◯ I took paid sick leave<br>◯ I did not need to take any sick leave<br>◯ I took unpaid leave because of illness as I do not have paid sick leave<br>◯ I had to leave my job or was let go because of illness

HEALTHCARE ACCESS

Did you try to get tested for COVID-19?	◯ Yes<br>◯ No
Have you ever had a positive test for COVID-19?	◯ Yes<br>◯ No, and I don't think I have had COVID-19<br>◯ No, but I think I had COVID-19
If you needed medical treatment for COVID-19 (e.g. see a doctor, get a chest x-ray, stay in the hospital, etc.), did you have access to the treatment you needed?	◯ Yes<br>◯ No<br>◯ Did not need treatment
How did you pay for the cost of your COVID-19 treatment?	◯ My insurance covered all the costs.<br>◯ My insurance paid for some of the costs and I paid a co-payment.<br>◯ I was responsible for the full amount of the treatment out-of-pocket.<br>◯ My employer paid for the treatment.<br>◯ Don't know.

FINANCES DURING COVID-19

How did your household income differ during the pandemic?	◯ No significant difference<br>◯ Had less income<br>◯ Had more income<br>(Think about all sources of money or other resources coming into the household during the stay at home period, including wages and unemployment benefits.)
How did your household expenses differ during the pandemic? Select all that apply.	☐ Expenses were lower<br>☐ Expenses stayed the same<br>☐ Expenses went up<br>☐ I accrued more debt than normal

Did you receive financial support from any of these sources within your social and family networks in the last 12 months?

	Yes	No	Don't know
Money from church or faith-based organization / community	○	○	○
Money from family or friends	○	○	○
Money from crowdsourcing platforms (e.g. GoFundMe)	○	○	○

Did you receive support from any of these government program sources in the last 12 months?

	Yes	No	Don't know
Unemployment insurance	◯	◯	◯
P-EBT (a second food benefit on your EBT card)	◯	◯	◯
Increased SNAP benefits	◯	◯	◯
Health insurance subsidy increase	◯	◯	◯
Deferment of student loan payments	◯	◯	◯
Child Tax Credit monthly payment	◯	◯	◯
Eviction prevention (also known as "eviction moratorium)	◯	◯	◯
Stimulus check	◯	◯	◯

How hard was it to get this support for your household?

◯ Extremely hard ◯ Somewhat hard ◯ Somewhat easy ◯ Very easy / Automatic

How helpful was this support for your household?

◯ Extremely helpful ◯ Somewhat helpful ◯ Not very helpful ◯ Not helpful at all

Tell us how these supports changed things for your household.

____

During the last 12 months, were your typical food shopping activities affected in any of the following ways?

	Yes	No
Unable to buy needed foods at grocery store (or other place you buy food) because food was out of stock	○	○
Affordable size or brand of food was not available	○	○
Food prices higher than usual	○	○
Closure of a grocery store or other place that you typically buy food at	○	○
Closure of a food shelf/pantry you typically rely on	○	○
Limited availability of foods at a food shelf/pantry you typically rely on	○	○
Started shopping for groceries online	○	○
Bought more take-out or prepared food than usual	○	○
Needed to buy more food than normal due to school closures	○	○
We were no longer able to choose our food at the food pantry/food shelf (i.e., we received a pre-packed bag, whereas we used to be able to choose)	○	○

2022 (T5) Wages Participant Survey

Please take this survey.
You may open the survey in your web browser by clicking the link below:

If the link above does not work, try copying the link below into your web browser:

This link is unique to you and should not be forwarded to others.

How many adults (age 18 or older) currently live in your household (including yourself)?

- ◯ 1
- ◯ 2
- ◯ 3
- ◯ 4
- ◯ 5 or more

How many children (under the age of 18) currently live in your household, including children who live there part-time?

- ◯ 0
- ◯ 1
- ◯ 2
- ◯ 3
- ◯ 4
- ◯ 5 or more

Please list the following information for each child living in the household:

Child 1
Age

____
(Years)

Child 1 Age

(Enter months if under age 1)

Years

____

Months

____

Child 1 Weight

____
(Pounds (lbs))

Child 1 Height

____
(Feet / Inches)

Child 2 Age

(Enter months if under age 1)

Years

____

Months

____

Child 2
Age

(Years)

Child 2 Weight

(Pounds (lbs))

Child 2 Height

(Feet / Inches)

Child 3
Age

(Years)

Child 3 Age

(enter months if under age 1)

Years

Months

Child 3 Weight

(Pounds (lbs))

Child 3 Height

(Feet / Inches)

Child 4
Age

(Years)

Child 4 Age

(Enter months if under age 1)

Years

Months

Child 4 Weight

(Pounds (lbs))

Child 4 Height

(Feet / Inches)

Child 5<br>Age	______________________<br>(Years)
Child 5 Age<br><br>(enter months if under age 1)	
Years	______________________
Months	______________________
Child 5 Weight	______________________<br>(Pounds (lbs))
Child 5 Height	______________________<br>(Feet / Inches)
How did you determine the child or children's height or weight?	◯ Pediatrician or health care provider records<br>◯ School records<br>◯ Measured at home<br>◯ My best guess<br>◯ Other
If you selected Other, please specify:	____________________________
Are you registered to vote?	◯ Yes<br>◯ No
Did you vote in the last local election, like for mayor or city council member?	◯ Yes<br>◯ No
Did you vote in the last general election, like for state representatives, senators and president?	◯ Yes<br>◯ No
During the past year did you attend a political protest, march or demonstration?	◯ Yes<br>◯ No
During the past year did you contact a public official?	◯ Yes<br>◯ No
During the past year did you work for a candidate or campaign?	◯ Yes<br>◯ No
In the last year (12 months), was there a time when you were not able to pay the mortgage or rent on time?	◯ Yes<br>◯ No

In the last year (12 months), how many places have you lived?

◯ 0
◯ 1
◯ 2
◯ 3
◯ More than 3

In the last year (12 months), was there a time when you did not have a steady place to sleep or slept in a shelter (including now)?

◯ Yes
◯ No

What is the highest grade or level of school you have completed?

◯ Less than High School
◯ Some High School
◯ High School Diploma
◯ Associate/Technical Degree
◯ Some College
◯ Bachelor's Degree
◯ Graduate Degree

When did you complete this schooling?

____
(Year (YYYY))

Have you completed any of the following types of trainings/career development activities in the last year (12 months)?
(Check all that apply)

☐ A training, workshop, seminar or professional event required by your employer
☐ A training, workshop, seminar or professional event not required by your employer
☐ Licensure or certification for your occupation
☐ A training, workshop, seminar or professional development event to help you change to a new occupation
☐ Other
☐ None of the above

If you selected Other, please specify:

____

What was your annual household income before taxes (from all sources), last year?

◯ Less than $5,000
◯ $5,001 to $10,000
◯ $10,001 to $20,000
◯ $20,001 to $30,000
◯ $30,001 to $40,000
◯ $40,001 to $50,000
◯ More than $50,000

Do you own or have access to a vehicle for reliable transportation?

◯ Yes
◯ No

In the last month (30 days) did you or anyone in your household receive any of the following? (Check Yes or No for EACH program)

	Yes	No	Not Sure
WIC (Women, Infants, and Children program)	◯	◯	◯
Food stamps (SNAP or EBT)	◯	◯	◯
Free or reduced price school lunch	◯	◯	◯
MFIP (Minnesota Family Investment Program) (You may receive one or more of the following together or separately: Cash, Food support, childcare assistance, housing grant.)	◯	◯	◯
State housing subsidy (e.g., Bridges Housing Subsidy, HUD Rental Assistance, Housing Choice/Housing Choice vouchers, Public Housing, Section 42 Housing)	◯	◯	◯
Disability assistance (including VA disability assistance)	◯	◯	◯
Work First (Temporary Assistance for the Needy Families, TANF)	◯	◯	◯

How much did your household receive in SNAP benefits in the last month (30 days)?

◯ I do not receive any food stamps or SNAP benefits
◯ $1 - $25
◯ $26 - $50
◯ $51 - $75
◯ $76 - $100
◯ $101 - $150
◯ $151 - $250
◯ $251 - $500
◯ $501 - $750
◯ More than $750

Please indicate your level of agreement with the following statements about using SNAP (or Food Stamp) food benefits since the COVID-19 pandemic?

(If you did not receive SNAP skip this question.)

Since the COVID-19 pandemic:

	Strongly disagree	Disagree	Neutral	Agree	Strongly agree	Don't know
Overall, it is not hard to use SNAP benefits to buy food for our household	◯	◯	◯	◯	◯	◯
SNAP benefits are not enough to meet our household's needs	◯	◯	◯	◯	◯	◯
We cannot use SNAP benefits to pay for groceries ordered online	◯	◯	◯	◯	◯	◯
We are not able to use our full months' worth of SNAP benefits (because, for example, it is hard to go shopping or stores do not have food we need)	◯	◯	◯	◯	◯	◯

Did your wages (dollars per hour) increase in the last year (12 months)?

◯ Yes
◯ No

If Yes, has your increase in wages made you worry about losing any public assistance? (housing subsidy, SNAP, WIC, MFIP, healthcare assistance)

◯ Yes
◯ No

Has your worry about losing assistance caused you to make job related changes? (quit job, asked to have hours reduced, got a different job)

◯ Yes
◯ No

Please explain the job changes you made because you were worried about losing public assistance.

____

Have you lost any public assistance in the last year (12 months)?

◯ Yes
◯ No

What public assistance did you lose in the last year (12 months)?

☐ WIC (Women, Infants, and Children program)
☐ Food stamps (SNAP)
☐ Free or reduced price school lunch
☐ MFIP (Minnesota Family Investment Program): Cash, Food support, childcare assistance, housing grant.)
☐ State housing subsidy (e.g., Bridges Housing Subsidy, HUD Rental Assistance, Housing Choice/Housing Choice vouchers, Public Housing, Section 42 Housing))
☐ Healthcare assistance
☐ Disability Assistance (including VA disability assistance)
☐ Work First (TANF)
☐ Other

If you selected Other, Please explain the public assistance you lost.	__________________________________
What happened that caused you to lose this assistance?	__________________________________
Do you have any other comments about using SNAP during the COVID-19 pandemic? (please specify)	__________________________________
What is your average monthly income from wages (i.e. pay for the jobs you work)?	◯ Less than $500<br>◯ $501 to $1,000<br>◯ $1,001 to $1,500<br>◯ $1,501 to $2,000<br>◯ $2,001 to $2,500<br>◯ More than $2,500
Do you have a physical health condition or disability that affects the number of hours of work you perform in a week?	◯ Yes<br>◯ No
Do you have mental health condition or disability that affects the number of hours of work you perform in a week?	◯ Yes<br>◯ No
Which one of the following best describes how well you are with managing money these days:	◯ Living comfortably<br>◯ Doing okay<br>◯ Just getting by<br>◯ Finding it difficult to get by
Compared to one year (12 months) ago, would you say that you (and your family living with you) are better off, the same, or worse off with money?	◯ Much better off<br>◯ Somewhat better off<br>◯ About the same<br>◯ Somewhat worse off<br>◯ Much worse off

Which of the following hardships did you or your family experience in the last year (12 months)? (Check all that apply)

- ☐ I lost a job
- ☐ I had my hours reduced
- ☐ I changed jobs
- ☐ I was suspended from work without pay
- ☐ I had a medical procedure or health emergency
- ☐ I reduced work hours or stopped working due to long-term health problems experienced after a COVID-19 infection (at least 4 weeks after infection)
- ☐ I experienced the death of another income earner
- ☐ I began caring for a loved one
- ☐ I was incarcerated
- ☐ I was arrested/charged with a crime
- ☐ I experienced divorce/separation
- ☐ I experienced the birth of a child
- ☐ I and/or my family lost transportation (vehicle repossessed or totaled, bus service cut etc...)
- ☐ I and/or my family experienced homelessness (homeless or displaced from home)
- ☐ I and/or my family received a foreclosure or eviction
- ☐ I and/or my family experienced the death of a loved one
- ☐ My partner/spouse lost their job
- ☐ My partner/spouse had their hours reduced
- ☐ Other
- ☐ None of the above

If you selected Other, please specify:

____

You responded above "I had a medical procedure or health emergency" - Was this related to COVID-19?	◯ Yes<br>◯ No
You responded above "I experienced the death of another income earner - Was this related to COVID-19?	◯ Yes<br>◯ No
You responded above "I began caring for a loved one" - Was this related to COVID-19?	◯ Yes<br>◯ No
You responded above "I experienced the death of a loved one" - Was this related to COVID-19?	◯ Yes<br>◯ No
In the last year (12 months), did you (or your husband/wife/partner) do any of the following? (check all that apply)	☐ Apply for a credit card or respond to a pre-approved credit card offer<br>☐ Request an increase in the credit limit of a credit card<br>☐ Apply for a mortgage or home-based loan<br>☐ Request to refinance a mortgage<br>☐ Apply for an auto loan<br>☐ Apply for a student loan<br>☐ Request an increase in the limit of an existing loan (other than a credit card)

How do you pay your bills? (Check all that apply)	☐ Electronic payment from bank<br>☐ Personal check<br>☐ Debit card<br>☐ Credit card<br>☐ Bank money order<br>☐ Cash<br>☐ Non-bank money order<br>☐ Pre-paid card<br>☐ Other
What other method do you use to pay your bills?	__________________________________
Have you used any of the following services in the last year (12 months)?<br>(Check all that apply)	☐ Pawn Shop<br>☐ Pay Day Loan<br>☐ Auto Title Loan<br>☐ Paycheck Advance Service<br>☐ Tax Refund Anticipation Loan<br>☐ Borrowed money from a friend, family or community member<br>☐ Got a loan from my employer<br>☐ None of the above
If you were faced with a $400 emergency expense today, how would you most likely cover it?<br><br>(Check all that apply)	☐ Use money from recent paycheck or savings<br>☐ Borrow money from a friend, family or community member<br>☐ Put it on my credit card and pay it off over time<br>☐ Sell something you own<br>☐ Delay payment on other bills or expenses<br>☐ Pawn Shop<br>☐ Pay Day Loan or Paycheck Advance Service<br>☐ Auto Title Loan<br>☐ Tax Refund Anticipation Loan<br>☐ Ask my employer for a loan<br>☐ I do not know how I would cover this expense<br>☐ Other
If you selected Other, Please explain the way you would cover this emergency expense	__________________________________
If you were faced with a $500 emergency expense today, how would you most likely cover it?<br><br>(Check all that apply)	☐ Use money from recent paycheck or savings<br>☐ Borrow money from a friend, family or community member<br>☐ Put it on my credit card and pay it off over time<br>☐ Sell something you own<br>☐ Delay payment on other bills or expenses<br>☐ Pawn Shop<br>☐ Pay Day Loan or Paycheck Advance Service<br>☐ Auto Title Loan<br>☐ Tax Refund Anticipation Loan<br>☐ Ask my employer for a loan<br>☐ I do not know how I would cover this expense<br>☐ Other
If you selected Other, Please explain the way you would cover this emergency expense	__________________________________

If you were faced with a $600 emergency expense today, how would you most likely cover it?<br><br>(Check all that apply)	☐ Use money from recent paycheck or savings<br>☐ Borrow money from a friend, family or community member<br>☐ Put it on my credit card and pay it off over time<br>☐ Sell something you own<br>☐ Delay payment on other bills or expenses<br>☐ Pawn Shop<br>☐ Pay Day Loan or Paycheck Advance Service<br>☐ Auto Title Loan<br>☐ Tax Refund Anticipation Loan<br>☐ Ask my employer for a loan<br>☐ I do not know how I would cover this expense<br>☐ Other
If you selected Other, please explain the way you would cover this emergency expense	__________________________________
In the last year (12 months), have you put off paying a bill because you didn't have enough money to pay it when it was due?	○ Yes<br>○ No
Do you have an account with a bank or credit union?	○ Yes<br>○ No

Part C. Accessing Food

What is the name and location of the store where MOST of the food for your household is purchased?

Name
Location (If you do not know the address you can list cross streets and nearby landmarks)
City

____

What is the name and location of the store where MOST of the food for your household is purchased?

Store Name: ______________________________

Store Location (address or nearby intersection, neighborhood, or landmark): ______________________________

Store Location (City): ______________________________

Who usually shops for most of the food for your household?

(Choose more than one if the task is evenly split)

- ☐ Me
- ☐ Spouse/Partner
- ☐ Child/Children
- ☐ Other adult in the home
- ☐ Other

If you selected Other, please specify: ______________________________

How do you usually get to the store where you get most of your food?

- ◯ Drive myself in a vehicle
- ◯ Family member/friends drive me in their vehicle
- ◯ Take public transportation (e.g. bus)
- ◯ Walk
- ◯ Taxi, Uber, or other car service
- ◯ Bicycle
- ◯ Other

If you selected Other, please specify: ______________________________

Please indicate how often each statement below was true for your household in the last year (12 months):

	Often true	Sometimes true	Never true
The food that we bought just didn't last and we didn't have money to get more.	◯	◯	◯
We couldn't afford to eat balanced meals.	◯	◯	◯

In the last year (12 months), did you or other adults in the household ever cut the size of your meal or skip meals because there was not enough money for food?

◯ Yes
◯ No
◯ Don't Know

How often did this happen?

◯ Almost every month
◯ Some months but not every month
◯ 1 or 2 months

In the last year (12 months), did you ever eat less than you felt you should because there wasn't enough money for food?

◯ Yes
◯ No
◯ Don't Know

In the last year (12 months), were you ever hungry but didn't eat because there wasn't enough money for food?

◯ Yes
◯ No
◯ Don't Know

In the last year (12-months) have you visited a food shelf (food bank or food pantry)?

◯ Yes
◯ No

Part D. Your Health

In general, would you say your health is:	◯ Excellent<br>◯ Very good<br>◯ Good<br>◯ Fair<br>◯ Poor
Are you currently pregnant?	◯ Yes<br>◯ No
Do you have health insurance? If so, please indicate what type(s) of health insurance you currently have.<br><br>(Check all that apply)	☐ Medicaid<br>☐ Medicare<br>☐ Health insurance plan offered through your employer<br>☐ Health insurance through your parent's insurance plan/employer<br>☐ Health insurance plan obtained through Healthcare.gov or MNsure (ACA Exchange)<br>☐ Private Insurance Plan (not obtained through insurance exchange/MNSure)<br>☐ Veterans' Administration (VA) Health Benefits<br>☐ Indian Health Services (IHS) tribal and/or urban Indian health programs<br>☐ Uninsured<br>☐ Other
If you checked Other, please specify:	__________________________________
When was the last time you visited a doctor, physician assistant or nurse in a clinic or medical office?<br><br>Do not include times you were hospitalized overnight or visits to the hospital emergency room.	◯ Within the last 12 months<br>◯ Within the last 1-2 years<br>◯ Within the last 2-5 years<br>◯ More than 5 years ago<br>◯ Never
In the last year (12 months), did you have an illness, injury, or condition that needed care right away in a clinic, emergency room, or doctor's office?	◯ Yes<br>◯ No
In the last year (12 months), when you needed care right away, how often did you get care as soon as you thought you needed it?	◯ Never<br>◯ Sometimes<br>◯ Usually<br>◯ Always
In the last year (12 months), not counting the times you needed care right away, did you make appointments for your health care at a doctor's office or clinic?	◯ Yes<br>◯ No
In the last year (12 months), how often did doctors or other health providers listen carefully to you?	◯ Never<br>◯ Sometimes<br>◯ Usually<br>◯ Always
In the last year (12 months), how often did doctors or other health providers show respect for what you had to say?	◯ Never<br>◯ Sometimes<br>◯ Usually<br>◯ Always

In the last year (12 months), how often did doctors or other health providers spend enough time with you?

- ◯ Never
- ◯ Sometimes
- ◯ Usually
- ◯ Always

Please check one of the boxes to indicate how strongly you agree or disagree with each statement.

	Strongly Disagree	Somewhat Disagree	Neutral	Somewhat Agree	Strongly Agree
I'm healthy enough that I really don't need health insurance.	◯	◯	◯	◯	◯
Health insurance is not worth the money it costs.	◯	◯	◯	◯	◯
I'm more likely to take risks than the average person.	◯	◯	◯	◯	◯
I can overcome illness without help from a medically trained person.	◯	◯	◯	◯	◯

Considering a 7-Day period (a week), how many times on average do you do the following kinds of exercise for more than 15 minutes during your free time:

STRENUOUS EXERCISE<br>(Heart beats rapidly)<br>Examples: running, jogging, hockey, football, soccer, basketball, cross-country skiing, roller blading, vigorous swimming, vigorous bicycling	______________________<br>(_____ times/week)
MODERATE EXERCISE<br>(Not exhausting)<br>Examples: fast walking, baseball, tennis, easy bicycling, badminton, easy swimming, downhill skiing, recreational dancing	______________________<br>(_____ times/week)
MILD EXERCISE<br>(Minimal effort)<br>Examples: easy walking, yoga, fishing, bowling, golf	______________________<br>(_____ times/week)
How much time do you usually spend sitting or reclining on a typical day?<br><br>Include time spent sitting at work, at home, getting to and from places (i.e. in a car, bus or train). Do not include time spent sleeping.	______________________<br>(Hours)
How much time did you spend last week playing video games?	______________________<br>(Hours)
What time do you usually go to bed (turn off the lights to sleep)?	◯ 1 AM ◯ 2 AM ◯ 3 AM<br>◯ 4 AM ◯ 5 AM ◯ 6 AM<br>◯ 7 AM ◯ 8 AM ◯ 9 AM<br>◯ 10 AM ◯ 11 AM ◯ 12 PM (noon)<br>◯ 1 PM ◯ 2 PM ◯ 3 PM<br>◯ 4 PM ◯ 5 PM ◯ 6 PM<br>◯ 7 PM ◯ 8 PM ◯ 9 PM<br>◯ 10 PM ◯ 11 PM ◯ 12 AM (midnight)

What time do you usually get out of bed?	◯ 1 AM ◯ 2 AM ◯ 3 AM<br>◯ 4 AM ◯ 5 AM ◯ 6 AM<br>◯ 7 AM ◯ 8 AM ◯ 9 AM<br>◯ 10 AM ◯ 11 AM ◯ 12 PM (noon)<br>◯ 1 PM ◯ 2 PM ◯ 3 PM<br>◯ 4 PM ◯ 5 PM ◯ 6 PM<br>◯ 7 PM ◯ 8 PM ◯ 9 PM<br>◯ 10 PM ◯ 11 PM ◯ 12 AM (midnight)
This question is about smoking status. Please select one of the following:	◯ Current smoker ◯ Quit less than 12 months ago ◯ Quit more than 12 months ago ◯ Never smoked
What product(s) do you smoke most regularly? (Check all that apply)<br><br>☐ Cigarettes<br>☐ Cigars, cigarillos, or little cigars<br>☐ E-cigarettes (Juul, e-cig, vaping pen)<br>☐ Tobacco in a hookah or waterpipe<br>☐ Pipes filled with tobacco (not waterpipes)<br>☐ Bidis (small brown cigarettes wrapped in a leaf)<br>☐ Marijuana<br>☐ Other	
If you selected Other, Please explain the other product you smoke?	__________________________________

Please indicate how often each statement below was true for you in the last month (30 days)

	Never	Almost Never	Sometimes	Fairly Often	Very Often
In the last month (30 days), how often have you felt unable to control the important things in your life?	○	○	○	○	○
In the last month (30 days), how often have you felt confident about your ability to handle your personal problems?	○	○	○	○	○
In the last month (30 days), how often have you felt that things were going your way?	○	○	○	○	○
In the last month (30 days), how often have you felt difficulties were piling up so high that you could not overcome them?	○	○	○	○	○

Patient Health Questionnaire-8

Now we're going to ask some questions about how you've been feeling lately. Some of these questions may be sensitive. Remember, you can skip any questions on this survey.

Over the last 2 weeks, how often have you been bothered by any of the following problems:

		Not at all	Several days	More than half the days	Nearly every day
1	Little interest or pleasure in doing things	○	○	○	○
2	Feeling down, depressed, or hopeless	○	○	○	○
3	Trouble falling or staying asleep, or sleeping too much	○	○	○	○
4	Feeling tired or having little energy	○	○	○	○
5	Poor appetite or overeating	○	○	○	○
6	Feeling bad about yourself, or that you are a failure or have let yourself or your family down	○	○	○	○
7	Trouble concentrating on things, such as reading the newspaper or watching television	○	○	○	○
8	Moving or speaking so slowly that other people could have noticed. Or the opposite - being so fidgety or restless that you have been moving around a lot more than usual	○	○	○	○

If answering any of these questions had made you feel like you'd like to talk to a professional, you can find someone to talk to by calling the National Suicide Prevention Lifeline at 800-273-8255

Please answer the following questions about your blood pressure, blood cholesterol and blood sugar.

Do you know your blood pressure?

If your response is "Yes", please provide the numbers below.

◯ Yes
◯ No

Diastolic Blood Pressure (bottom or second number):

____
(mmHG (50-120+))

Systolic Blood Pressure (top or first number):

____
(mmHG (80-210+))

Please respond to the following list of statements about your blood pressure. Check all that apply.

☐ In the past 12 months, a doctor, a nurse, or other health professional told you that you have high blood pressure.
☐ I take medication for high blood blood pressure
☐ I prefer not to answer

Cholesterol

Do you know your blood cholesterol level?

◯ Yes
◯ No

Please tell us your total blood cholesterol.

Total Blood Cholesterol (mg/dL (40-400+):

____

Please respond to the following statements about your cholesterol. Check all that apply.

☐ In the past 12 months, a doctor, a nurse, or other health professional told you that you have high cholesterol
☐ I take medication for high cholesterol
☐ I prefer not to answer

Do you know your blood sugar?

◯ Yes
◯ No

Please tell us your fasting blood sugar in the space provided.

Fasting Blood Sugar:

____
(mg/dL (50-500+))

Please respond to the following statements about your blood sugar. Check all that apply.

- ☐ In the past 12 months, a doctor, a nurse, or other health professional told you that you have high blood sugar
- ☐ I take blood sugar medication
- ☐ I have diabetes (either type I or II)
- ☐ I prefer not to answer

Part E. Dietary Screener Questionnaire

These questions are about foods you ate or drank during the past month (30 days). When answering, please include meals and snacks at home, at work or school, in restaurants and anyplace else.

During the past month (30 days), how often did you eat hot or cold cereals?	◯ Never ◯ 1 time last month<br>◯ 2-3 times last month<br>◯ 1 time per week ◯ 2 times per week<br>◯ 3-4 times per week ◯ 5-6 times per week ◯ 1 time per day<br>◯ 2 or more times per day
During the past month (30 days), what kind of cereal did you usually eat? (For example, Honey Nut Cheerios, grits, oatmeal, Cinnamon Toast Crunch, Froot Loops, etc. Please be as DETAILED as possible.)	______________________________
If there was another kind of cereal that you usually ate during the past month (30 days), what kind was it?	______________________________<br>(Skip if no other kind of cereal)
During the past month (30 days), how often did you drink regular soda or pop that contains sugar? Do not include diet soda.	◯ Never ◯ 1 time last month<br>◯ 2-3 times last month<br>◯ 1 time per week ◯ 2 times per week<br>◯ 3-4 times per week ◯ 5-6 times per week ◯ 1 time per day<br>◯ 2-3 times per day ◯ 4-5 times per day<br>◯ 6 or more times per day
During the past month (30 days), how often did you drink 100% pure fruit juices such as orange, mango, apple, grape and pineapple juices?<br><br>Do not include fruit-flavored drinks with added sugar or fruit juice you made at home and added sugar to.	◯ Never ◯ 1 time last month<br>◯ 2-3 times last month<br>◯ 1 time per week ◯ 2 times per week<br>◯ 3-4 times per week ◯ 5-6 times per week ◯ 1 time per day<br>◯ 2-3 times per day ◯ 4-5 times per day<br>◯ 6 or more times per day
During the past month (30 days), how often did you drink coffee or tea that had sugar or honey added to it?<br><br>Include coffee and tea you sweetened yourself and presweetened tea and coffee drinks such as Arizona Iced Tea and Frappuccino. Do not include artificially sweetened coffee or diet tea.	◯ Never ◯ 1 time last month<br>◯ 2-3 times last month<br>◯ 1 time per week ◯ 2 times per week<br>◯ 3-4 times per week ◯ 5-6 times per week ◯ 1 time per day<br>◯ 2-3 times per day ◯ 4-5 times per day<br>◯ 6 or more times per day
During the past month (30 days), how often did you drink sweetened fruit drinks, sports or energy drinks, such as Kool-Aid, lemonade, Hi-C, cranberry drink, Gatorade, Red Bull or Vitamin Water?<br><br>Include fruit juices you made at home and added sugar to. Do not include diet drinks or artificially sweetened drinks.	◯ Never ◯ 1 time last month<br>◯ 2-3 times last month<br>◯ 1 time per week ◯ 2 times per week<br>◯ 3-4 times per week ◯ 5-6 times per week ◯ 1 time per day<br>◯ 2-3 times per day ◯ 4-5 times per day<br>◯ 6 or more times per day

During the past month (30 days), how often did you eat fruit?<br><br>Include fresh, frozen, or canned fruit. Do not include juices.	◯ Never ◯ 1 time last month<br>◯ 2-3 times last month<br>◯ 1 time per week ◯ 2 times per week<br>◯ 3-4 times per week ◯ 5-6 times per week ◯ 1 time per day<br>◯ 2 or more times per day
During the past month (30 days), how often did you eat a green leafy or lettuce salad, with or without other vegetables?	◯ Never ◯ 1 time last month<br>◯ 2-3 times last month<br>◯ 1 time per week ◯ 2 times per week<br>◯ 3-4 times per week ◯ 5-6 times per week ◯ 1 time per day<br>◯ 2 or more times per day
During the past month (30 days), how often did you eat any kind of fried potatoes, including French fries, home fries, or hash brown potatoes?	◯ Never ◯ 1 time last month<br>◯ 2-3 times last month<br>◯ 1 time per week ◯ 2 times per week<br>◯ 3-4 times per week ◯ 5-6 times per week ◯ 1 time per day<br>◯ 2 or more times per day
During the past month (30 days), how often did you eat any other kind of potatoes, such as baked, boiled, mashed potatoes, sweet potatoes, or potato salad?	◯ Never ◯ 1 time last month<br>◯ 2-3 times last month<br>◯ 1 time per week ◯ 2 times per week<br>◯ 3-4 times per week ◯ 5-6 times per week ◯ 1 time per day<br>◯ 2 or more times per day
During the past month (30 days), how often did you eat refried beans, baked beans, beans in soup, pork and beans or any other type of cooked dried beans?<br><br>Do not include green beans.	◯ Never<br>◯ 1 time last month<br>◯ 2-3 times last month<br>◯ 1 time per week<br>◯ 2 times per week<br>◯ 3-4 times per week<br>◯ 5-6 times per week<br>◯ 1 time per day<br>◯ 2 or more times per day
During the past month (30 days), how often did you eat brown rice or other cooked whole grains, such as bulgur, cracked wheat, or millet? Do not include white rice.	◯ Never ◯ 1 time last month<br>◯ 2-3 times last month<br>◯ 1 time per week ◯ 2 times per week<br>◯ 3-4 times per week ◯ 5-6 times per week ◯ 1 time per day<br>◯ 2 or more times per day
During the past month (30 days), not including the foods you just reported on (i.e., green salads, potatoes, cooked dried beans), how often did you eat other vegetables?	◯ Never ◯ 1 time last month<br>◯ 2-3 times last month<br>◯ 1 time per week ◯ 2 times per week<br>◯ 3-4 times per week ◯ 5-6 times per week ◯ 1 time per day<br>◯ 2 or more times per day

During the past month (30 days), how often did you have Mexican-type salsa made with tomato?	◯ Never ◯ 1 time last month ◯ 2-3 times last month ◯ 1 time per week ◯ 2 times per week ◯ 3-4 times per week ◯ 5-6 times per week ◯ 1 time per day ◯ 2 or more times per day
During the past month (30 days), how often did you eat pizza? Include frozen pizza, fast food pizza, and homemade pizza.	◯ Never ◯ 1 time last month ◯ 2-3 times last month ◯ 1 time per week ◯ 2 times per week ◯ 3-4 times per week ◯ 5-6 times per week ◯ 1 time per day ◯ 2 or more times per day
During the past month (30 days) how often did you eat any type of fish or other seafood such as canned tuna, baked or fried fish of any type, or shellfish like shrimp or crab?	◯ Never ◯ 1 time last month ◯ 2-3 times last month ◯ 1 time per week ◯ 2 times per week ◯ 3-4 times per week ◯ 5-6 times per week ◯ 1 time per day ◯ 2 or more times per day
During the past month (30 days), how often did you have tomato sauces such as with spaghetti or noodles or mixed into foods such as lasagna? Do not include tomato sauce on pizza.	◯ Never ◯ 1 time last month ◯ 2-3 times last month ◯ 1 time per week ◯ 2 times per week ◯ 3-4 times per week ◯ 5-6 times per week ◯ 1 time per day ◯ 2 or more times per day
During the past month (30 days), how often did you eat whole grain bread including toast, rolls and in sandwiches? Whole grain breads include whole wheat, rye, oatmeal and pumpernickel. Do not include white bread.	◯ Never ◯ 1 time last month ◯ 2-3 times last month ◯ 1 time per week ◯ 2 times per week ◯ 3-4 times per week ◯ 5-6 times per week ◯ 1 time per day ◯ 2 or more times per day
During the past month (30 days), how often did you eat chocolate or any other types of candy? Do not include sugar-free candy.	◯ Never ◯ 1 time last month ◯ 2-3 times last month ◯ 1 time per week ◯ 2 times per week ◯ 3-4 times per week ◯ 5-6 times per week ◯ 1 time per day ◯ 2 or more times per day
During the past month (30 days), how often did you eat doughnuts, sweet rolls, Danish, muffins, pan dulce, or pop-tarts? Do not include sugar-free items.	◯ Never ◯ 1 time last month ◯ 2-3 times last month ◯ 1 time per week ◯ 2 times per week ◯ 3-4 times per week ◯ 5-6 times per week ◯ 1 time per day ◯ 2 or more times per day

During the past month (30 days), how often did you eat cookies, cake, pie, or brownies? Do not include sugar-free kinds.	◯ Never ◯ 1 time last month ◯ 2-3 times last month ◯ 1 time per week ◯ 2 times per week ◯ 3-4 times per week ◯ 5-6 times per week ◯ 1 time per day ◯ 2 or more times per day
During the past month (30 days), how often did you eat ice cream or other frozen desserts? Do not include sugar-free kinds.	◯ Never ◯ 1 time last month ◯ 2-3 times last month ◯ 1 time per week ◯ 2 times per week ◯ 3-4 times per week ◯ 5-6 times per week ◯ 1 time per day ◯ 2-3 times per day ◯ 4-5 times per day ◯ 6 or more times per day
During the past month (30 days), how often did you eat popcorn?	◯ Never ◯ 1 time last month ◯ 2-3 times last month ◯ 1 time per week ◯ 2 times per week ◯ 3-4 times per week ◯ 5-6 times per week ◯ 1 time per day ◯ 2-3 times per day ◯ 4-5 times per day ◯ 6 or more times per day
General Survey Notes	______________________________

Part F. Household Spending

About how much did your household spend in the last month (30 days) on the following? Enter $0 if you did not spend any money in that category, Enter N/A if this category does not apply to you.

Mortgage (including taxes, interest and principal) __________________________________

Rent __________________________________

Electricity __________________________________

Water __________________________________

Heating fuel for the house __________________________________

Cable, internet, and land line phone payment amount __________________________________

Cell phone __________________________________

Groceries __________________________________

Other food (restaurants, take-out) __________________________________

The next section is about transportation spending.

The next section is about Transportation spending.

About how much did your household spend in the last month (30 days) on the following? Enter $0 if you did not spend any money in that category. Enter N/A if this category does not apply to you.

Car or vehicle payments (interest and principal) ______________________

Car or vehicle insurance ______________________

Gasoline ______________________

Public transit ______________________

Other transportation expenses (parking, tolls, taxi, and rideshares apps like Uber) ______________________

Healthcare spending

About how much did your household spend in the last month (30 days) on the following? Enter $0 if you did not spend any money in that category. Enter N/A if this category does not apply to you.

Healthcare spending
About how much did your household spend in the last month (30 days) on the following?
Enter $0 if you did not spend any money in that category. Enter N/A if this category does not apply to you.

Health insurance premium ______________________________

Prescription and nonprescription medications (Include only out-of-pocket expenses. Do not include what is covered by insurance) ______________________________

Nursing home care or at-home care service (Include only out-of-pocket expenses. Do not include what is covered by insurance) ______________________________

Eye care (Include only out-of-pocket expenses. Do not include what is covered by insurance) ______________________________

Dental care (Include only out-of-pocket expenses. Do not include what is covered by insurance) ______________________________

Other medical expenses, such as copays (Include only out-of-pocket expenses. Do not include what is covered by insurance) ______________________________

Exercise/sports (including gym, exercise equipment such as bicycles, skis, etc.) ______________________________

Childcare (including daycare, babysitters, and afterschool care) ______________________________

Cash or gifts to family or friends outside the household (including "tithes" or donations to churches/synagogues/mosques) ______________________________

Savings ______________________________

Child support ______________________________

Student loan payments ______________________________

Did you purchase any of the following in the last year (12 months)?<br><br>Include newly purchased items not fully paid for at the time of purchase.<br><br>Check all that apply	☐ Automobile or truck<br>☐ Major vehicle repair<br>☐ Large home appliance (refrigerator, stove/oven, dishwasher, washing machine, dryer, etc.)<br>☐ Major home repair (furnace, roof, walls, etc.)<br>☐ Major home furniture (couch/sofa, bed, etc.)<br>☐ Television<br>☐ Computer<br>☐ Trip or vacation<br>☐ None of the above
Spending Notes	__________________________________
Mortgage=	____________________________
Rent=	____________________________
Electricity=	____________________________
Water=	____________________________
Heating=	____________________________
Cable/Internet/Land line phone=	____________________________
Cell phone=	____________________________
Groceries=	____________________________
Other Food=	____________________________
Car payment=	____________________________
Car insurance=	____________________________
Gasoline=	____________________________
Public transit=	____________________________
Other transportation=	____________________________

Health insurance= ________________________

Medications= ________________________

Nursing home/at-home care= ________________________

Eye care= ________________________

Dental care= ________________________

Exercise= ________________________

Childcare= ________________________

Cash/gifts to others= ________________________

Savings= ________________________

Child support= ________________________

Student loan payments= ________________________

Part G. Self-reported Weight

Note: If we are not able to measure height in Year 3 and Year 4 we will use the Year 2 value as the default.

What is your current weight?

____
(Based on your best estimate)

Height measurement from last in-person visit:
[height_2]

Part H. Pandemic Impact

We will ask a number of questions about your experience during the pandemic.

Which of these options best describe your employment situation during the last 12 months?	◯ Unemployed throughout this period<br>◯ Worked at same job/employer throughout this period<br>◯ Got a new job<br>◯ Lost my job<br>◯ Quit my job (e.g. because I didn't feel safe)
Did you experience any of the following during the last 12 months? Select all that apply.	☐ Reduction in hours worked<br>☐ Increase in hours worked<br>☐ Reduction in hourly wage or salary<br>☐ Increase in hourly wage or salary (e.g., hazard pay)<br>☐ Furlough (Defined as temporary leave of absence after which your employer wants you back in your position .)<br>☐ Laid off<br>☐ None of the above
Did your employer take measures to protect you from health risks due to the pandemic?	◯ Yes<br>◯ No<br>((i.e. Did your workplace make changes such as installing plastic barriers for cashiers, provision of face masks, surgical gloves, or other coverings for employees, or implement other measures?))
Did you have access to paid sick leave as part of your compensation and benefits from your employer?	◯ Yes<br>◯ No<br>◯ Partially / Some of the time<br>◯ Do not know
Did you take paid leave because you were sick or had to take care of someone who was sick?	◯ I took paid sick leave<br>◯ I did not need to take any sick leave<br>◯ I took unpaid leave because of illness as I do not have paid sick leave<br>◯ I had to leave my job or was let go because of illness
Have you ever had a positive test for COVID-19?	◯ Yes<br>◯ No, and I don't think I have had COVID-19<br>◯ No, but I think I had COVID-19
If you needed medical treatment for COVID-19 (e.g. see a doctor, get a chest x-ray, stay in the hospital, etc.), did you have access to the treatment you needed?	◯ Yes<br>◯ No<br>◯ Did not need treatment
How did you pay for the cost of your COVID-19 treatment?	◯ My insurance covered all the costs.<br>◯ My insurance paid for some of the costs and I paid a co-payment.<br>◯ I was responsible for the full amount of the treatment out-of-pocket.<br>◯ My employer paid for the treatment.<br>◯ Don't know.

FINANCES DURING COVID-19

How did your household income differ during the pandemic?	◯ No significant difference<br>◯ Had less income<br>◯ Had more income<br>(Think about all sources of money or other resources coming into the household during the stay at home period, including wages and unemployment benefits.)
How did your household expenses differ during the pandemic? Select all that apply.	☐ Expenses were lower<br>☐ Expenses stayed the same<br>☐ Expenses went up<br>☐ I accrued more debt than normal

Did you receive financial support from any of these sources within your social and family networks in the last 12 months?

	Yes	No	Don't know
Money from church or faith-based organization / community	○	○	○
Money from family or friends	○	○	○
Money from crowdsourcing platforms (e.g. GoFundMe)	○	○	○

Did you receive support from any of these government program sources in the last 12 months?

	Yes	No	Don't know
Unemployment insurance	◯	◯	◯
P-EBT (a second food benefit on your EBT card)	◯	◯	◯
Increased SNAP benefits	◯	◯	◯
Health insurance subsidy increase	◯	◯	◯
Deferment of student loan payments	◯	◯	◯
Child Tax Credit monthly payment	◯	◯	◯
Eviction prevention (also known as "eviction moratorium")	◯	◯	◯
Emergency Rental Assistance (RentHelpMN or HOPE)	◯	◯	◯

How hard was it to get this support for your household?

◯ Extremely hard ◯ Somewhat hard ◯ Somewhat easy ◯ Very easy / Automatic

How helpful was this support for your household?

◯ Extremely helpful ◯ Somewhat helpful ◯ Not very helpful ◯ Not helpful at all

Tell us how these supports changed things for your household.

____

During the last 12 months, were your typical food shopping activities affected in any of the following ways?

	Yes	No
Unable to buy needed foods at grocery store (or other place you buy food) because food was out of stock	○	○
Affordable size or brand of food was not available	○	○
Food prices higher than usual	○	○
Closure of a grocery store or other place that you typically buy food at	○	○
Closure of a food shelf/pantry you typically rely on	○	○
Limited availability of foods at a food shelf/pantry you typically rely on	○	○
Started shopping for groceries online	○	○
Bought more take-out or prepared food than usual	○	○
Needed to buy more food than normal due to school closures	○	○
We were no longer able to choose our food at the food pantry/food shelf (i.e., we received a pre-packed bag, whereas we used to be able to choose)	○	○

If you responded Other, please specify details: ______________________________
